# Effects of acute and chronic oxidative stress on the blood–brain barrier in 2D and 3D in vitro models

**DOI:** 10.1186/s12987-022-00327-x

**Published:** 2022-05-12

**Authors:** Tracy D. Chung, Raleigh M. Linville, Zhaobin Guo, Robert Ye, Ria Jha, Gabrielle N. Grifno, Peter C. Searson

**Affiliations:** 1 Institute for Nanobiotechnology, Johns Hopkins University, 100 Croft Hall, 3400 North Charles Street, Baltimore, MD USA; 2 Department of Biomedical Engineering, Johns Hopkins University, Baltimore, MD USA; 3 Department of Applied Mathematics and Statistics, Johns Hopkins University, Baltimore, MD USA; 4 Department of Materials Science and Engineering, Johns Hopkins University, Baltimore, MD USA

**Keywords:** Blood–brain barrier, Oxidative stress, Brain microvascular endothelial cells, Hydrogen peroxide, Barrier function

## Abstract

Oxidative stress is a shared pathology of neurodegenerative disease and brain injuries, and is derived from perturbations to normal cell processes by aging or environmental factors such as UV exposure and air pollution. As oxidative cues are often present in systemic circulation, the blood–brain barrier (BBB) plays a key role in mediating the effect of these cues on brain dysfunction. Therefore, oxidative damage and disruption of the BBB is an emergent focus of neurodegenerative disease etiology and progression. We assessed barrier dysfunction in response to chronic and acute oxidative stress in 2D and 3D in vitro models of the BBB with human iPSC-derived brain microvascular endothelial-like cells (iBMECs). We first established doses of hydrogen peroxide to induce chronic damage (modeling aging and neurodegenerative disease) and acute damage (modeling the response to traumatic brain injury) by assessing barrier function via transendothelial electrical resistance in 2D iBMEC monolayers and permeability and monolayer integrity in 3D tissue-engineered iBMEC microvessels. Following application of these chronic and acute doses in our in vitro models, we found local, discrete structural changes were the most prevalent responses (rather than global barrier loss). Additionally, we validated unique functional changes in response to oxidative stress, including dysfunctional cell turnover dynamics and immune cell adhesion that were consistent with changes in gene expression.

**Supplementary Information:**

The online version contains supplementary material available at 10.1186/s12987-022-00327-x.

## Background

Oxidative stress is caused by an imbalance of reactive oxygen species (ROS) and other oxidants in relation to antioxidant systems that sequester these species to maintain homeostasis [1, 2]. ROS are capable of chemically disrupting the structure of lipids, proteins, and DNA, ultimately damaging cells and tissues, and hence oxidative stress is implicated in a wide variety of diseases and injury in nearly every organ system [3, 4]. Additionally, oxidative stress and its correlated diseases are often most prominent in the aging population, although environmental cues, such as air pollution and UV exposure, play a significant role in the acceleration of these diseases in all age groups [5–7]. ROS include hydroxyl radicals, superoxide ions, and hydrogen peroxide (H_2_O_2_), which are produced endogenously in homeostasis but become overabundant in oxidative stress [4].

Of particular interest is the role that oxidative stress plays in neurodegenerative diseases (NDD) where increased oxidative stress is linked to severity of disease pathology [8–11]. Biomarkers of oxidative stress (e.g., peroxiredoxins and ubiquinone/ubiquinol) are elevated in individuals with various NDDs including Alzheimer’s disease, Parkinson’s disease, and amyotrophic lateral sclerosis, and are correlated with cognitive impairments [12–14]. In aging and in response to environmental cues, ROS are not only derived from the brain itself, but from systemic exposure [15–18]. Therefore, the blood–brain barrier (BBB) plays a key role in ROS-mediated injury and diseases of the brain. The BBB tightly regulates transport into and out of the brain via tight junctions, transport systems, and efflux proteins, and is thought to be a key link between vascular comorbidities (e.g., coronary artery disease and diabetes) and NDDs, a concept that is commonly referred to as the vascular hypothesis [19–23]. Immunohistochemical staining of post-mortem human brain tissue and in vivo dye-injection studies in mice have found that local disruption of the BBB due to ROS exposure is correlated with inflammation of the surrounding brain tissue in individuals with NDD [24] and in animal models [25]. Importantly, the susceptibility of the BBB to ROS-induced dysfunction may play a key role in initiation and progression of NDDs [26].

However, while the studies described above have been key in establishing the links between oxidative stress, the BBB, and neurodegenerative disease, a detailed and systematic understanding of BBB dysfunction under oxidative conditions is limited. Many studies rely on staining post-mortem tissue (in which functionality cannot be further studied) [24], intrinsic or extrinsic animal studies (in which species-specific differences may dominate) [25, 27, 28], or exposure of 2D cell monolayers to oxidative agents (e.g., H_2_O_2_) in which microenvironmental cues are absent [20, 29, 30]. In addition, the pathological effects of oxidative stress are dose-dependent, incorporating both concentration and exposure duration, but many studies do not explore these factors. Acute profiles, which represent a shorter but more intense period of oxidative stress, are associated with traumatic brain injury and ischemia–reperfusion in stroke [31, 32]. For example, studies in rat models of cerebral ischemia/reperfusion have shown a sixfold increase in oxidative stress-responsive apoptosis inducing protein (ORAIP) in cerebrospinal fluid within 30 min of injury, then returns to control levels after four hours [33]. On the other hand, chronic profiles, which represent a longer but less intense period of oxidative stress, are more common in neurodegenerative disease and aging [34–37].

Some early studies in chip-based BBB models have also modeled oxidative and nitrosative stress, primarily via prodrugs such as linsidomine or menadione, with a particular focus on rescuing bulk barrier function following concentration-dependent dosing [38, 39]. To advance our understanding on a more granular level, here we utilize human induced pluripotent stem cell (iPSC)-derived brain microvascular endothelial-like cells (iBMECs) in 2D monolayers and 3D tissue-engineered microvessel models of the BBB that recapitulate the physiological geometry, shear cues, and barrier function of native tissue [40, 41] to identify differences in acute and chronic stress responses to the physiologically-produced ROS hydrogen peroxide (H_2_O_2_). In vitro models were exposed to H_2_O_2_ concentrations spanning three orders of magnitude across short and long exposure times to identify chronic and acute profiles for further study. These representative profiles resulted in the generation of discrete, local defects which are easily identifiable in tissue-engineered iBMEC microvessels with high spatiotemporal resolution. Additional transcriptomic and functional assessments of these profiles indicated cell cycle disruption and inflammatory immune cell responses that are unique to either chronic or acute oxidative stress. These studies deepen our insight into the dynamic role of oxidative stress in injury and disease of the BBB.

## Materials and methods

### Cell culture

Human induced pluripotent stem cell-derived brain microvascular endothelial-like cells (iBMECs) recapitulate key properties of human BMECs, including expression of tight junctions, efflux pumps, and nutrient transporters [42, 43]. Differentiation of iBMECs was performed as previously reported [44]. iBMECs were differentiated from three isogenic iPSC cell lines from a healthy donor (Allen Cell Collection, WTC parent), that express fluorescently-labeled zona occludens-1 (ZO1), beta-actin (ACTB), and plasma membrane, which produce iBMECs with near-identical morphology and transendothelial electrical resistance [39]. After eleven days of differentiation, iBMECs were detached using Accutase (Thermo Fisher Scientific, A1110501) and seeded onto Transwell inserts or type I collagen microvessel channels coated with 50 µg mL^−1^ human placental collagen IV (Sigma-Aldrich, cat. no. C5533) and 25 µg mL^−1^ fibronectin from human plasma (Sigma Aldrich, cat. no. F2006). During seeding and for the following 24 h, cells were cultured in BBB induction medium composed of human endothelial cell serum-free medium (HESFM) (Thermo Fisher Scientific, cat. no. 11111044) supplemented with 1% human serum from platelet-poor plasma (Sigma-Aldrich, cat. no. P2918), 1% penicillin–streptomycin (Thermo Fisher Scientific, cat. no. 15140122), 2 ng mL^−1^ human recombinant basic fibroblast growth factor (bFGF; Fisher Scientific, cat. no. 233FB025CF), and 10 µM all-trans retinoic acid (Sigma-Aldrich, cat. no. R2625). For 24 h before H_2_O_2_ exposure, cells were further cultured in BBB maintenance medium: HESFM supplemented with 1% human serum from platelet-poor plasma and 1% penicillin–streptomycin.

In 2D Transwell and 3D microvessel experiments, iBMECs were exposed to different concentrations of hydrogen peroxide (H_2_O_2_) for 1 h (acute) or over a period of 10 days (chronic). Different concentrations of H_2_O_2_ were prepared by diluting the stock solution (Sigma Aldrich, H1009) in BBB maintenance medium. In all experiments, we define the exposure as the initial concentration at the beginning of the experiment. To determine the concentration profile of H_2_O_2_ in medium over time, we measured H_2_O_2_ levels using the Amplex Red Hydrogen Peroxide Kit (Thermo Fisher, A22188).

### Transwell-based TEER, cell count, and ROS assays

Transwell inserts (Corning, cat. no. 3470) were coated with 50 µg mL^−1^ collagen IV and 25 µg mL^−1^ fibronectin overnight at 37 °C, then seeded with iBMECs in BBB induction medium at 1 × 10^6^ cells cm^−2^. After 24 h, the medium was switched to BBB maintenance medium and daily transendothelial electrical resistance (TEER) measurements were performed using an EVOM-2 and STX-100 electrodes (World Precision Instruments, Sarasota, FL). TEER measurements were recorded daily before initiating experiments or changing medium; monolayers with TEER > 1500 Ω cm^2^ during the 24 h of culture in BBB maintenance media (day 0) were used for experiments. Three experimental conditions were distributed across Transwell inserts: (1) control in BBB maintenance medium without any media switches (control), (2) exposure to sterile water (vehicle), or (3) exposure to various concentrations of H_2_O_2_. All exposures were conducted by adding 5 µL of water or H_2_O_2_ to the apical chamber (representing a 20 × dilution). For chronic exposure, confluent monolayers in Transwells were treated with H_2_O_2_ on day 0 and TEER was measured daily for ten days with no media changes. For acute exposure, confluent monolayers in Transwells were treated with H_2_O_2_ on day 0 for 30–60 min, and then the media was replaced with fresh BBB maintenance medium (no H_2_O_2_); TEER was then measured every hour for the four subsequent hours and also daily for ten days. All TEER values are reported after subtracting the value for a blank transwell and correcting for the insert area.

To measure intracellular ROS levels and cell counts, monolayers on inserts were washed twice with phosphate buffered saline (PBS; Gibco, 10010049) and treated with Cellular Reactive Oxygen Species Detection Assay Kit (Deep Red Fluorescence) (Abcam, ab186029) according to manufacturer protocols and 40 µg mL^−1^ DAPI solution (Sigma-Aldrich, D9542), respectively. These measurements were conducted at various time points after seeding (days 1, 2, and 4) following a one-hour incubation at 37 °C. Images were collected via epifluorescence on an inverted microscope (Nikon Eclipse TiE) using a 4 × objective and NIS Advanced Research software (Nikon). The resultant images were processed using CellProfiler, utilizing built-in modules of illumination correction and identification of primary objects to yield cell count, and utilizing mask generation of cell areas and measuring corresponding intensities to yield ROS signal per cell [45].

### Tissue-engineered BBB model

Three-dimensional in vitro blood–brain barrier microvessels were generated as previously reported [46]. Briefly, polydimethylsiloxane (PDMS; Dow Corning, 4019862) housings were molded with a cavity for hydrogel patterning and plasma-bonded to glass slides. Utilizing a 150 µm diameter nitinol wire (McMaster-Carr, 8320K12), 1 cm long channels (total surface area of ~ 0.05 cm^2^) were patterned in a rectangular prism of 7 mg mL^−1^ type 1 collagen (Corning, 354249). Wires were then removed from the hydrogel leaving an empty channel. 20 mM genipin (Wako Biosciences, MFCD00888600) was perfused through channels for two hours to stiffen collagen and increase cell adhesion [46]. Channels were incubated with matrix proteins (50 µg mL^−1^ type IV collagen and 25 µg mL^−1^ fibronectin), and seeded with iBMECs suspended in BBB induction medium, supplemented with 10 µM Rho-associated protein kinase (ROCK) inhibitor Y27632 (ATCC, ACS-3030) at a concentration of 5 × 10^6^ cells mL^−1^ under extremely low-flow conditions (~ 0.01 mL h^−1^) and allowed to adhere for 30 min before initiation of perfusion. Sterile, chemically inert, and noncytotoxic reservoirs (Fisher Scientific, 14-817-32) were connected to both inlet and outlet ports. Perfusion through the vessel was established by filling the upstream reservoir with medium to a height of 5 cm, resulting in a volumetric flow rate of ~ 0.5 mL h^−1^. The channels were perfused with BBB induction medium supplemented with ROCK inhibitor for 24 h, resulting in the formation of confluent microvessels, and then switched to BBB maintenance medium, and maintained under steady flow. After 24 h in BBB maintenance medium, confluent microvessels were perfused with BBB maintenance medium supplemented with 100 µM H_2_O_2_ continuously (chronic exposure), perfused with BBB maintenance medium supplemented with 500 µM H_2_O_2_ for one hour followed by perfusion with unsupplemented BBB maintenance medium (acute exposure), or corresponding vehicle control.

To ensure that oxygen depletion during perfusion of the microvessels did not contribute to oxidative stress, we measured the levels of dissolved oxygen in the medium in the inlet reservoir and in the perfusate from the outlet over 5 days using a dissolved oxygen probe (RCYAGO, DO9100).

### Measurement of permeability and identification of defects

To assess changes in permeability and defect formation, microvessels were perfused with two fluorescent solutes: 2 µM Alexa Fluor647-conjugated 10 kDa dextran (Thermo Fisher Scientific, cat. no. D22914) and 200 µM Lucifer yellow (LY; Sigma, CH dilithium salt) in BBB maintenance medium. Microvessels were then imaged (Nikon Eclipse TiE) at 10 × magnification and maintained in an environmental chamber at 37 °C. Epifluorescence illumination was controlled by X-Cite 120LEDBoost (Excelitas Technologies). Phase contrast images (assembled from images from 10 adjacent frames) were acquired every two minutes at the longitudinal top plane, midplane, and bottom plane of the microvessel; fluorescence images were acquired every 2 min at the microvessel midplane. Microvessels were imaged for 10 min before and 1 h following perfusion with the fluorescent solutes.

Three types of structural defects were identified manually by assessing each cropped phase and fluorescent image: (1) delaminations with no focal leak, (2) focal leaks with no delamination, and (3) combination focal leaks and delaminations. Delaminations were identified from phase images where the midplane of the vessel endothelium showed detachment from the channel wall, focal leaks were identified from fluorescence images where there was visible localized leakage of fluorescent solute from the vessel lumen, and combined defects were identified by overlaying the phase and fluorescent images. The time to defect resolution was determined by monitoring fluorescent solute leakage over image sequences.

### Bulk RNA-sequencing and gene set enrichment analysis

RNA was collected from 2D monolayers of iBMECs seeded onto Transwells in the presence of ROCK inhibitor, to match experimental conditions used in tissue-engineered iBMEC microvessels. Collection occurred immediately or four days after acute (500 µM) exposure to H_2_O_2_ for 1 h and their corresponding vehicle controls, or seven days after initiation of chronic (100 µM) exposure to H_2_O_2_ and its corresponding vehicle control. Cells were washed with PBS and then lysed using RLT buffer supplemented with 1% β-mercaptoethanol (Sigma, M3148). RNA was purified using the RNeasy Mini Kit (Qiagen) with DNase I digestion, and eluted in RNase-free water, following kit protocols. All sequencing was performed by Novogene on an Illumina NovaSeq platform with paired end 150 bp reads, generating approximately 20 million paired reads per sample. cDNA libraries were constructed following oligo (dT) capture and enrichment. All samples had an RNA integrity number > 8.8 (Agilent 2100 Bioanalyzer).

Raw read alignment and quantification to the reference human genome (GRCh38) was performed using the R (v4.0.1) package Rsubread (v2.0.1) [48]. Normalization, visualization, and differential analysis was performed using the R package DESeq2 (v1.28.1) [49]. Normalization of raw reads in DESeq2 utilized variance stabilizing regularized logarithm transformation (rlog) before calculation of Euclidean sample distances and resultant principal component analysis (PCA). Differentially expressed genes (DEGs) were identified using the Wald test with Benjamini–Hochberg correction-adjusted p-values, where values < 0.05 were considered statistically significant. Gene set enrichment analysis was conducted by inputting an enriched set of genes, composed of all DEGs and genes with > 3 log_2_FC, into Enrichr and assessing GO biological processes and MSigDB Hallmark pathways [50]. Visualizations (PCA, volcano plots) were formatted using R package ggplot2 (v3.3.2) [51].

### Cell turnover analysis

Cell loss and mitosis events in microvessels were counted manually from phase contrast imaging sequences acquired every 2 min over 1 h. The total number of iBMECs were counted manually from the polar planes of the microvessel, then cell loss and mitosis events were observed manually, as reported previously [39]. Briefly, cell loss events were identified by an appearance of cell swelling and disappearance from the monolayer. Mitosis events were identified by observing cell compression and formation of daughter cells. The rate of turnover was calculated from the difference between the rates of mitosis and cell loss, and is presented as a percentage of all cells per hour (% h^−1^).

### Immune cell adhesion

Immune cell adhesion assays were conducted at days 1 and 4 after H_2_O_2_ exposure in a tissue-engineered microvessel. Human THP-1 cells, a monocytic cell line derived from an acute monocytic leukemia patient, were cultured in RPMI-1640 medium (Thermo Fisher Scientific, 11875093) supplemented with 10% fetal bovine serum (Sigma, F4135) and 1% penicillin–streptomycin (Thermo Fisher Scientific, cat. no. 15140122). An expanded stock of THP-1s were frozen in liquid nitrogen and thawed 24 h prior to use. Before each experiment, THP-1s were suspended in serum-free RPMI-1640 medium and incubated for 20 min with CellTracker Deep Red (Thermo Fisher Scientific, C34565) according to product protocols. After washing the cells twice with PBS, THP-1s were resuspended at a final concentration of 1 × 10^6^ cells mL^−1^ and 70 µL of this suspension (70,000 cells) was perfused through the device for 10 min under low shear stress (~ 0.2 dyne cm^−2^). Afterwards, any remaining cell suspension upstream of the device was removed and replaced with BBB maintenance medium to wash out non-adherent THP-1s for 20 min. Adherent fluorescently-labeled THP-1s were manually counted in each device, and normalized to microvessel length.

### Statistical analysis

Biological replicates are defined as experiments conducted with an independent differentiation of iBMECs. All statistical analysis was performed using Prism 9 (GraphPad). All data are presented as mean ± SEM (standard error of the mean). A student’s unpaired *t*-test (two-tailed with unequal variances) was used for comparison of two groups and an analysis of variance (ANOVA) was used for comparisons of three or more groups. Tukey’s multiple comparisons test was used to compare experimental conditions across groups of three or more, where reported p-values were multiplicity adjusted. Statistical significance was defined at *p* < 0.05, with *p < 0.05, **p < 0.01, and ***p < 0.001.

## Results

**Fig. 1:**
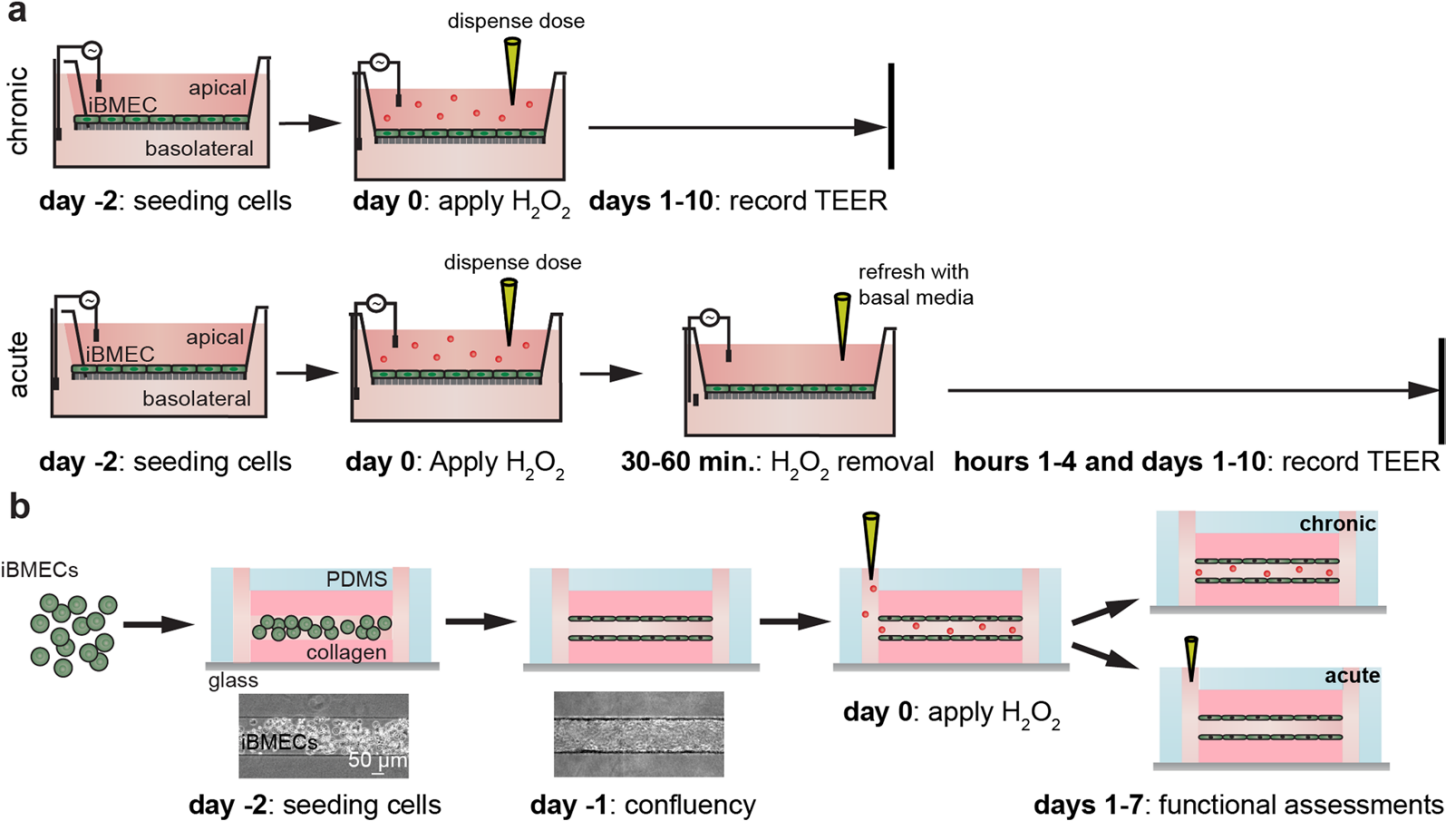
Methods to model oxidative stress in 2D and 3D. **a** Schematic depicting experimental protocol of exposing 2D iBMEC monolayers on Transwells to chronic and acute doses of H_2_O_2_. **b** Schematic depicting seeding of iBMECs into 3D microchannels, formation of BBB microvessels, and exposure to chronic and acute H_2_O_2_

Neurodegenerative diseases and brain injuries are associated with acute or chronic exposure to oxidative stress. Since there are no established protocols for modeling oxidative stress exposure, we first determined conditions for acute and chronic injury of iBMEC monolayers to the ROS hydrogen peroxide (H_2_O_2_). H_2_O_2_ is a commonly studied oxidative agent used in vitro due to its capacity to mimic oxidative stress by stimulating hydroxyl radical formation and its relative stability in cell culture medium [52]. A key challenge for in vitro studies is modeling a pathological response to mimic human disease and injury. Results from animal studies have indicated that baseline levels of H_2_O_2_ may be around 50 µM, although following injury, such as ischemia–reperfusion in the striata of rat brains, levels may increase to 200 µM [53]. Although there have been relatively few studies of cytotoxic exposure to H_2_O_2_ in humans, levels of systemic irritation are known to occur at around 100 µM when inhaled and 10 mM when ingested, which provide benchmarks for exposure in our model systems [54–56]. Based on these observations, we sought to establish models of chronic and acute oxidative stress exposure in 2D iBMEC monolayers on Transwells (Fig. 1a) and in 3D tissue-engineered iBMEC microvessels (Fig. 1b).

To establish dose regimes leading to physiological, pathological, and cytotoxic responses, iBMEC monolayers in Transwells and microvessels were exposed to concentrations of H_2_O_2_ from 10 µM to 10 mM for durations of up to 1 h (acute exposure) or up to 10 days (chronic exposure). From independent measurements of the H_2_O_2_ concentration in cell culture over time, we found that the concentration of H_2_O_2_ decreased over several days (Additional file 1: Fig. S1). Therefore, the H_2_O_2_ concentration in acute exposure is approximately constant, whereas in chronic exposure the H_2_O_2_ levels decrease over time, more significantly after 5 days of culture.

### In vitro measurements recapitulate acute and chronic oxidative stress profiles

**Fig. 2:**
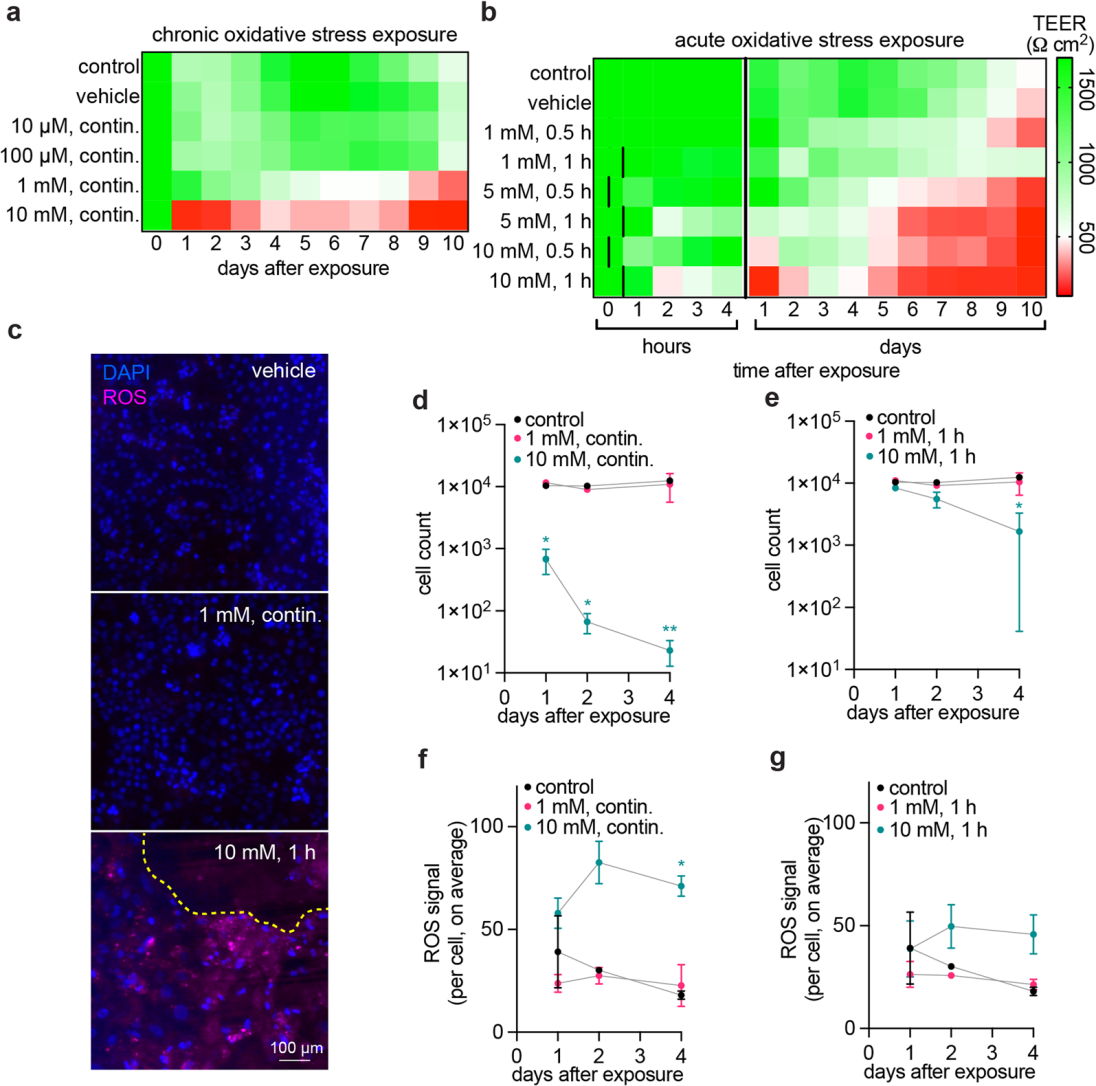
Dose-dependent response of 2D iBMEC monolayers to chronic and acute oxidative stress. **a** Heatmap of iBMEC TEER during chronic exposure to H_2_O_2_ at various concentrations and corresponding controls (no addition, or vehicle addition). Continuous 1 mM H_2_O_2_ exposure resulted in a gradual loss of barrier function over 10 days that models chronic oxidative stress. Each cell represents the mean of three technical replicates across *n* = 3 biological replicates in all conditions. **b** Heatmap of iBMEC TEER during acute exposure to H_2_O_2_ at various concentrations and exposure times and corresponding controls (no addition, or vehicle addition). 10 mM H_2_O_2_ exposure for one hour resulted in significant but recoverable loss of barrier function over 10 days that is consistent with acute oxidative stress. Each cell represents the mean of three technical replicates across *n* = 7 biological replicates in all conditions. **c** Representative images from DAPI and ROS assay staining showing the negative control with minimal ROS accumulation, continuous 1 mM H_2_O_2_ exposure with minimal ROS accumulation, and acute 10 mM H_2_O_2_ exposure with visible ROS accumulation and cell loss. Yellow dotted line indicates a region of cell loss (no nuclei). **d**, **e** Quantification of cell counts over time following chronic and acute exposure, respectively. **f**, **g** Quantification of intracellular ROS per cell over time following chronic and acute exposure, respectively. Statistical tests were conducted versus control at each timepoint. *n* = 3 biological replicates for all conditions and time courses for (**d–g**)

To establish the influence of H_2_O_2_ dose on barrier function, we first assessed changes in TEER in 2D confluent monolayers of iBMECs (Fig. 2a, b). In agreement with previous human exposure studies, continuous exposure to 10 µM or 100 µM resulted minimal changes in TEER over 10 days (Fig. 2a). Continuous exposure to 1 mM H_2_O_2_ resulted in a gradual decrease in TEER over several days, with values below 500 Ω cm^2^ on day 9 after exposure (*p = 0.018 vs. vehicle). TEER values for iBMECs below 500 Ω cm^2^ are associated with increased solute permeability [57]. Acute doses probed the interactions between H_2_O_2_ concentration and exposure duration, spanning 1 mM to 10 mM exposure for either 30 or 60 min (Fig. 2b). An exposure of 1 mM H_2_O_2_ for one hour resulted in a gradual decrease in TEER beginning on day two and decreasing to 700 Ω cm^2^ over 10 days, but not significantly different from control conditions (ns, *p* = 0.836). However, a ten-fold increase in concentration to 10 mM H_2_O_2_ for one hour dramatically decreased TEER within three hours (*p = 0.013 vs. vehicle), with values remaining low for the following two days (*p = 0.036 vs. vehicle), before returning to values similar to controls on day three following exposure (ns, *p* = 0.633 vs. vehicle). This profile is consistent with acute and reversible damage that recovers over several days. To understand the role of cellular damage in these TEER studies, we further investigated cell counts and intracellular ROS levels (Additional file 1: Fig. S2c–g). While measurable ROS levels were correlated with macroscopic and functional disruption of the monolayer, oxidative stress responses were below the detection limit of the assay. These observations are exemplified in representative images of a negative vehicle control, 1 mM chronic H_2_O_2_ exposure that produced no measurable ROS but resulted in TEER loss, and 10 mM acute H_2_O_2_ exposure that resulted in measurable ROS and significant cell loss (i.e., toxicity) (Fig. 2c). Cell counts remained unchanged in chronic and acute exposure to 1 mM H_2_O_2_, but resulted in dramatic decreases in cell count during continuous 10 mM exposure (*p = 0.018, 0.016, and **p = 0.001 vs. control at days 1, 2, and 4, respectively), and significant decrease in cell count by day 4 in acute one-hour 10 mM exposure (*p = 0.032 vs. control) (Figs. 2d, e). ROS accumulation was measured using a fluorescent detection kit and followed similar trends to cell count (Additional file 1: Fig. S1e, f). Chronic and acute 1 mM H_2_O_2_ exposure produced no measurable change in ROS accumulation, but continuous 10 mM exposure resulted in a significant increase in ROS by day four (*p = 0.022 vs. control) (Fig. 2f). Acute one-hour 1 mM H_2_O_2_ exposure resulted in slight increases in ROS, but not at a significant level (*p* = 0.117 vs. control at day 4) (Fig. 2g). In summary, ROS were detected only at cytotoxic exposures associated with significant cell loss from the monolayer. At lower concentrations, we observed decreased TEER values but the sensitivity of the assay was not sufficient to detect increases in ROS.

Together, these results are consistent with competition between homeostatic forces, stress exposure, and the capacity of antioxidants to regulate ROS accumulation and promote proliferation to maintain a confluent monolayer. There are three key parameters in selecting dosing conditions to model chronic and acute oxidative stress: (1) exposure should be below previously documented concentrations that trigger systemic irritation (which includes presentation of burning of the eyes or skin, itching and dryness in the throat, cough, and a variety of other symptoms) in humans (10 mM), (2) exposure should recapitulate measurable barrier dysfunction, and (3) exposure should not result in significant cell death. Therefore, the dosing range of the perturbation should lie in a “pathological” regime: maintenance of relatively high cell viability but sustaining some loss of barrier function. The pathological response should be between “physiological” (maintenance of high cell viability and no evidence of loss of barrier function) and “cytotoxic” (very low cell viability with significant loss of barrier function). Our 2D experiments suggest that the pathological stress regime, prior to the onset of cytotoxicity, may be relatively narrow in vitro; thus, we selected the following conditions to model acute and chronic oxidative stress exposure: (1) chronic oxidative stress: continuous 1 mM H_2_O_2_ exposure, resulting in a gradual decrease in TEER, and (2) acute oxidative stress: a one-hour exposure to 10 mM H_2_O_2_, resulting in a short-term decrease and partial recovery in TEER.

### Three-dimensional models reveal discrete, local defects in response to oxidative stress

**Fig. 3:**
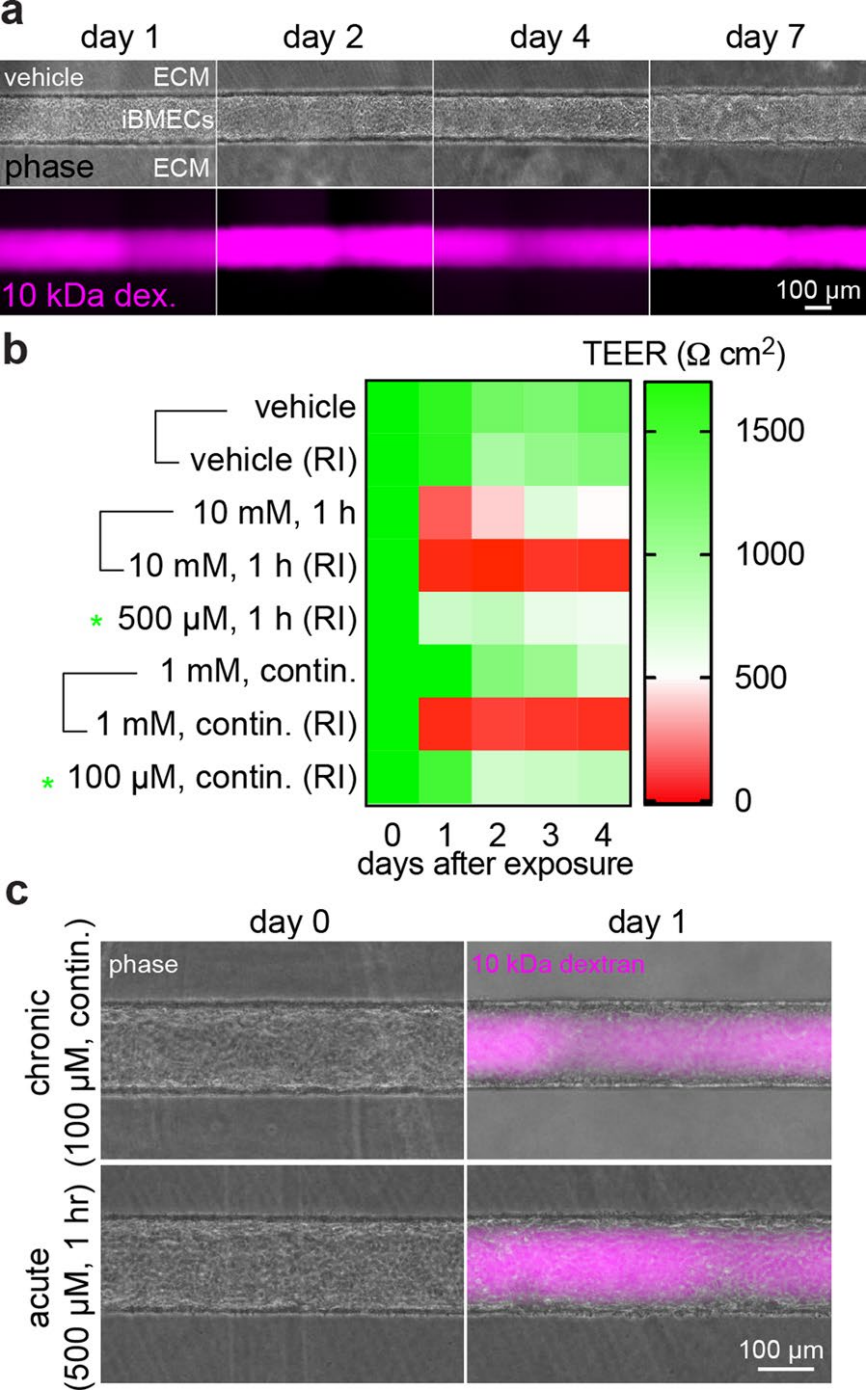
Response to chronic and acute oxidative stress in a tissue-engineered iBMEC microvessel. **a** Phase contrast and fluorescence images of a control microvessel (treated with vehicle) during 1 h perfusion with tagged fluorescently-labeled 10 kDa dextran. **b** Heatmap demonstrating the influence of ROCK inhibitor (RI) on TEER in 2D studies. The paired conditions demonstrate that RI sensitizes iBMEC monolayers to oxidative stress. Green asterisks indicate selected conditions for acute and chronic exposure in the presence of RI to further explore in 3D microvessels. Each cell represents the mean of three technical replicates across *n* = 3 biological replicates in all conditions. **c** Representative phase and phase/fluorescence overlays during perfusion with 10 kDa dextran at chronic and acute doses of H_2_O_2_ showing maintenance of monolayer confluency and dye restriction

To model oxidative stress in a system mimicking the physiological cues of the BBB microenvironment, we next performed experiments in a tissue-engineered iBMEC microvessel model perfused with culture media supplemented with H_2_O_2_ (Fig. 1b). Microvessels maintained barrier function for at least one week as previously reported [40], and remained confluent over at least seven days after maturation with no focal leaks (Fig. 3a). The 3D microenvironment and cell culture conditions maintained normal oxygen exchange and cellular respiration. The dissolved oxygen in the inlet reservoir was on average 17.7%, close to the expected value of 18.6% under normal cell culture conditions (5 vol.% CO_2_ and 37 °C) (Additional file 1: Fig. S3) [47]. The concentration in the outlet perfusate was on average 16.8%, indicating a ~ 0.9% drop in dissolved oxygen due to cell metabolism during perfusion. However, there are two major differences between 2D and 3D culture conditions relevant to oxidative stress exposure. First, 3D microvessels are continually perfused with ROS in comparison to monolayers in 2D Transwells. Second, 3D microvessels are perfused with medium supplemented with Rho-associated protein kinase (ROCK) inhibitor (Y27632) during seeding and the first 24 h of perfusion (to enhance cell adhesion and spreading). Therefore, expected conditions to recapitulate acute and chronic stress exposure are different in 3D than in 2D systems (Additional file 1: Fig. S4a, b).

To determine these different exposures, we conducted additional experiments in 2D monolayers to evaluate the influence of ROCK inhibitor on iBMEC responses to oxidative stress (Fig. 3b). When added to the media in Transwells prior to H_2_O_2_ exposure, ROCK inhibitor resulted in a significant decrease in TEER; therefore, reduced H_2_O_2_ doses best recapitulated chronic and acute oxidative stress dynamics given these culture conditions (Fig. 3b, Additional file 1: Fig. S4c). In 3D, these corresponding reduced H_2_O_2_ doses resulted in confluent monolayers and minimal change in global permeability immediately following exposure (Fig. 3c). However, local dysfunction emerged on longer time-scales, which we explored by perfusion with fluorescently tagged 10 kDa dextran and Lucifer yellow (MW 444.24 Da).

**Fig. 4:**
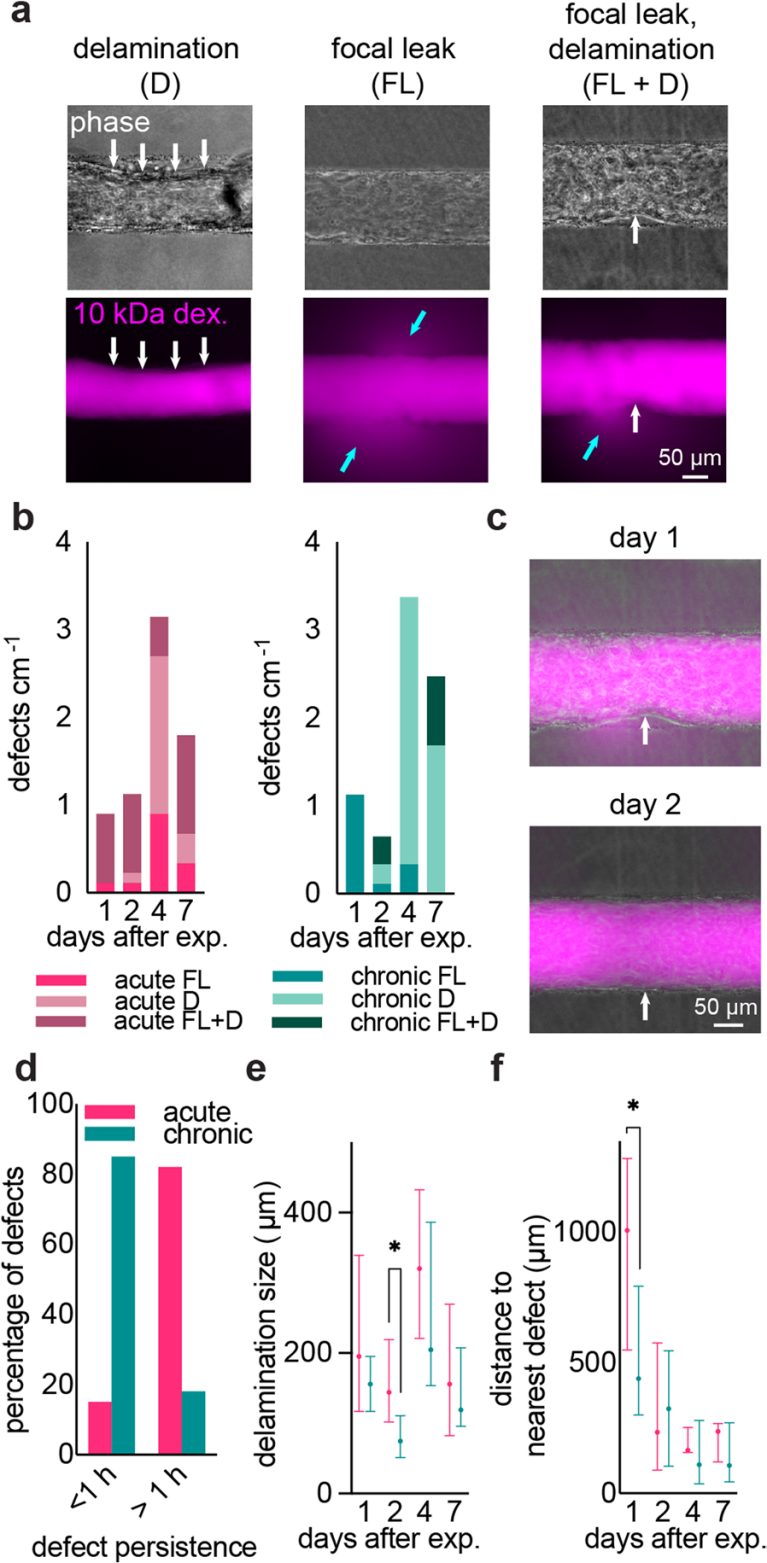
Characterization of local defects in tissue-engineered iBMEC microvessels in response to chronic and acute oxidative stress. **a** Representative examples of the three defect types: delamination, focal leak, and combination focal leak/delamination. White arrows indicate delaminations and blue arrows indicate focal leaks. **b** Distribution of defect types in chronic and acute exposure over time. **c** Representative images showing resolution of a delamination defect over one day following acute exposure. **d** Histogram of persistence of delaminations under chronic and acute stress exposure. **e**, **f** Distribution of delamination sizes and distance to nearest defect under chronic and acute exposure to oxidative stress over time. Data (**b**, **d**–**f**) represents *n* = 124 defects across both conditions with *n* = 3 independent microvessels for each condition

From phase contrast images and fluorescence images, we found that discrete, local defects dominated over global changes to permeability. Defects were characterized as focal leaks, delaminations (local loss of adhesion), or combination focal leaks with delaminations (Fig. 4a). All three types of defects were observed with both chronic and acute exposure, and the number of unique defects increased with time (Fig. 4b). Comparing chronic and acute exposure, combination focal leak/delaminations were more prevalent in acute conditions, with this trend becoming more significant over time (**p = 0.005 at day 4, ***p < 0.001 at day 7). Additionally, while the distribution of defect type within acute conditions did not favor a particular defect classification, chronic conditions resulted in significantly more delaminations than focal leaks or combination focal leak/delaminations, with the comparative significance peaking at day 4 after exposure (**p = 0.007 for delaminations vs. focal leaks, **p = 0.008 for delaminations vs. combination focal leak/delaminations). While defect distribution was dependent on oxidative stress condition, in confluent regions away from defect sites, the local permeability of fluorescent solutes was largely unchanged (Additional file 1: Fig. S5), which indicates that discrete defects are the primary sites of dysfunction and that barrier function elsewhere in the microvessels is unaffected.

From daily imaging of microvessels we observed that all three types of defects had the potential for recovery (Fig. 4c). Delaminations resolved by re-adherence of the endothelium to the ECM, while focal leaks resolved by reversal of dye leakage. To understand the time course of this recovery, we defined the persistence as the duration of time between the appearance of the defect and re-adherence (for delaminations) and resolution of dye leakage (for focal leaks). Acute exposure resulted in increased persistence of defects, whereas chronic exposure resulted in defects that typically recovered on the order of minutes (Fig. 4d).

Delaminations were generally larger and exhibited a broader range of sizes following acute exposure. Chronic exposure resulted in smaller delaminations (with the difference between conditions peaking at day 2), although the range of sizes increased with time (Fig. 4e). Defect clustering was assessed from the distance to the nearest defect and, for both acute and chronic exposure, delaminations became more clustered several days after initial exposure, with the only significant difference occurring one day after exposure (Fig. 4f).

### Bulk RNA-sequencing highlights upregulation of inflammatory pathways following oxidative stress

**Fig. 5:**
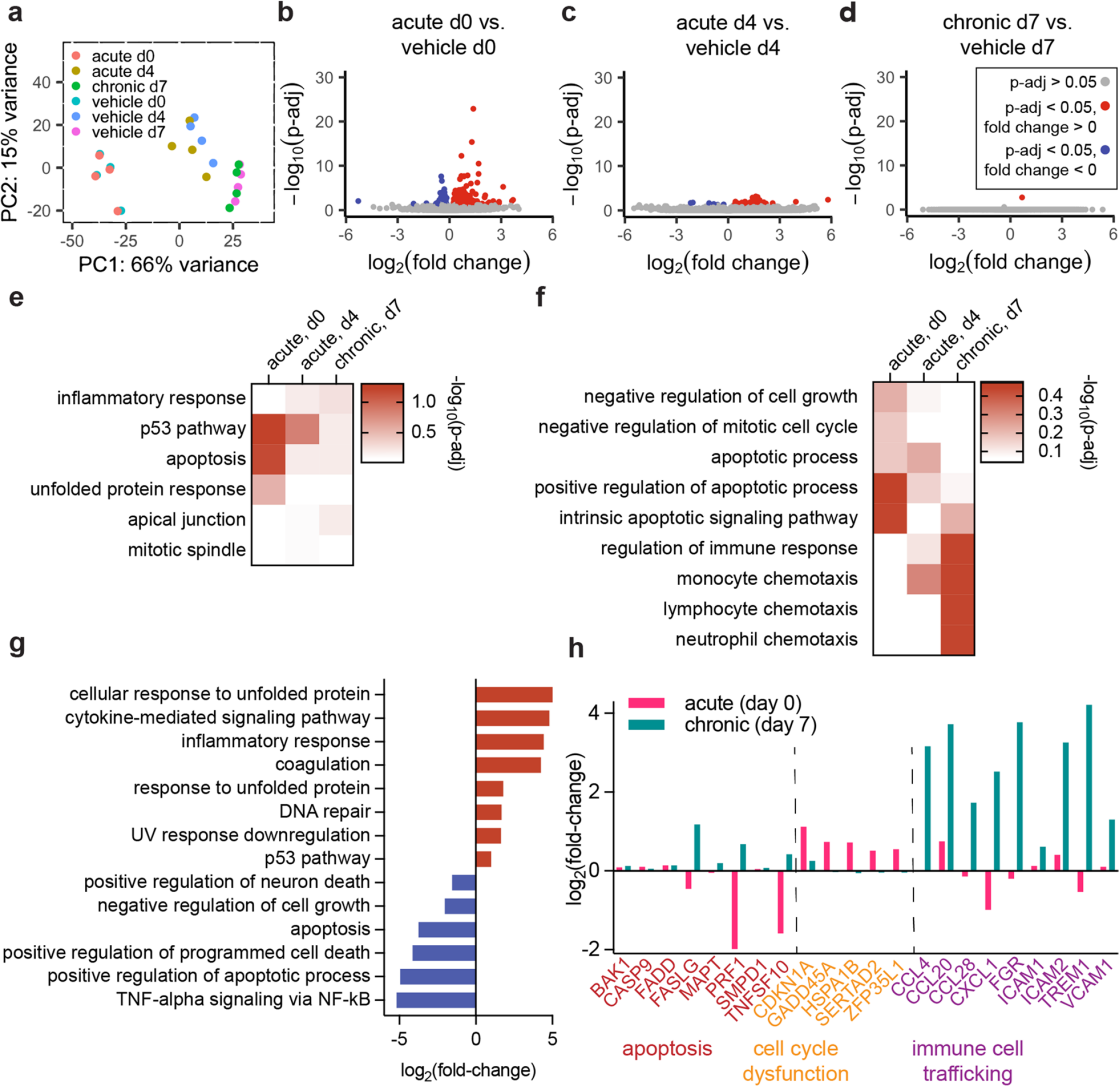
Chronic and acute oxidative stress modulate iBMEC gene expression. **a** Principal component analysis of RNA collected from iBMECs seeded on Transwells in the presence of ROCK inhibitor one hour after acute exposure (d0), four days after acute exposure (d4), and seven days after chronic exposure (d7), and their respective vehicle controls. Each sequencing sample is pooled across two technical replicates for *n* = 4 biological replicates for each condition and time-point. **b–d** Volcano plots of differentially expressed genes in oxidatively stressed samples compared to their respective vehicle controls at days 0, 4, and 7 after exposure. **e**–**f** Heatmaps of adjusted p-value of selected pathways of MSigDB Hallmark Pathway and GO Biological Processes terms, respectively, from genes with log_2_(fold-change) > 3. **g** Fold-change comparison of key pathways from GSEA indicating up- or down-regulation at day 4 after acute exposure vs. hour 1 after acute exposure. Pathways highlighted here were selected by sorting all resultant pathways by combined score (top 10 for MSigDB Hallmark and top 50 for GO Biological Processes). Pathways where one time-point had a combined score of zero were excluded. **h** Comparison of enriched genes, specific to key pathways for functional analysis, showing unique enrichment in acute or chronic oxidative stress profiles

Having characterized the structural changes that lead to loss of barrier function, we next assessed gene expression to identify other aspects of BBB function altered by oxidative stress. To simultaneously verify barrier function via TEER measurements (Additional file 1: Fig. S6a), and to collect enough nucleic acid material for sequencing, 3D exposure conditions were matched in 2D Transwell conditions and 3D iBMEC microvessels were utilized to validate and visualize oxidative stress responses found in transcriptomic results. iBMECs were seeded in the presence of ROCK inhibitor onto transwells and treated with either control medium, medium supplemented with 100 µM H_2_O_2_, or medium supplemented with 500 µM H_2_O_2_ which was then replaced with control medium after one hour (matching 3D conditions explored in Figs. 3 and 4). RNA was collected immediately after acute exposure, four days after acute exposure, and seven days after chronic exposure. In all cases, RNA from control monolayers was collected at identical timepoints for paired comparisons. From principal component analysis of the results, we found that maturation time had a larger effect on gene expression than oxidative stress experimental conditions (Fig. 5a). These results indicate that iBMECs in 2D monolayers undergo substantial phenotypic drift that should be addressed in future work.

To explore differences between oxidative stress conditions, we identified differentially expressed genes (DEGs) (Fig. 5b–d) and conducted gene set enrichment analysis (GSEA) with all genes with a log_2_(fold-change) > 3 for all experimental conditions (Fig. 5e, f). Immediately after acute exposure we found 175 upregulated and 116 downregulated genes (p-adj < 0.05) compared to the control. Four days after acute exposure, the number of upregulated and downregulated DEGs decreased to 39 and 6, respectively. Interestingly, seven days after chronic exposure, there was only one upregulated gene compared to control. The fold-change of key genes related to the highlighted pathways reflected these patterns: generally, genes related to BBB identity and function were downregulated, while genes related to inflammation, protein breakdown, and immune cell adhesion were upregulated (Additional file 1: Fig. S6b). Selected genes associated with active barrier transport (such as efflux pumps and receptor-mediated endocytosis proteins) were also assessed (Additional file 1: Fig. S6c) and most transporters showed changes in expression levels of log_2_(fold-change) < 1.

The top upregulated molecular pathways across all oxidative stress exposures from MSigDB Hallmark (Fig. 5e, Additional file 1: Fig. S6d) and GO Biological Processes families (Fig. 5f, Additional file 1: Fig. S6e) were predominantly associated with inflammation and survival, including shared enriched pathways in both exposure schemes related to programmed cell death (MSigDB: Apoptosis, GO: 0006915, 0043065, 0097193, 0070059, 1902043, 0043281, 0001844, 0043068), p53 pathways (MSigDB: p53 Pathway), and specific inflammatory pathways, including tumor necrosis factor and subsequent NF-κB signaling (MSigDB: TNF-alpha Signaling via NF-kB, GO: 0071346), which provide important targets for designing assays of these cytokine pathways during BBB dysfunction. Additionally, key oxidative stress response genes, particularly glutaredoxins and glutathione peroxidases, stress fiber formation genes, and key Intermediate-Early Genes (IEGs) are upregulated among all oxidative stress conditions (Additional file 1: Figs. S7, S8c). Enriched pathways unique to acute oxidative stress included multiple gene sets associated with negative regulation of homeostatic processes and cell cycle arrest (GO: 0032845, 0045926, 0030308, 0045930). Enriched pathways unique to chronic oxidative stress involved immune cell attraction and adhesion (GO: 0050776, 0060099, 0050764, 0050855, 0043304, 0002548, 0048247, 0070098, 0030593, 2000403, 0051133). Both general families of upregulated pathways provide insight into functional changes that were further studied in our in vitro system.

Comparison of transcriptomic changes on day four compared to immediately after acute exposure were consistent with observed functional recovery in 2D monolayers two days after exposure to H_2_O_2_ (Fig. 5g). A fold-change comparison of top MSigDB Hallmark and GO Biological Processes between four days after exposure and one hour after exposure revealed a time-dependent downregulation in many inflammatory and apoptotic pathways (MSigDB: Apoptosis, TNF-alpha Signaling via NF-kB, GO: 0030308, 0043068, 1901216), and upregulation of cellular stress responses, such as DNA repair (MSigDB: DNA Repair) and coagulation (MSigDB: Coagulation). This shift suggests barrier recovery is concurrent with a decrease in inflammatory responses.

GSEA revealed a number of shared pathways between chronic and acute oxidative stress, but to demonstrate the utility of our in vitro models in representing unique modes of dysfunction, we sought pathways that were unique to either exposure profile to pursue in functional assays. Pathway-level comparisons indicated changes in apoptotic, mitotic, and other cell cycle changes were more prevalent in acute oxidative stress, while upregulation of immune cell chemotaxis and migration was more prevalent in chronic oxidative stress. Additionally, families of associated genes for each pathway more specifically reflect these trends (Fig. 5h).

### Chronic and acute oxidative stress modulate cell turnover and immune cell adhesion

**Fig. 6:**
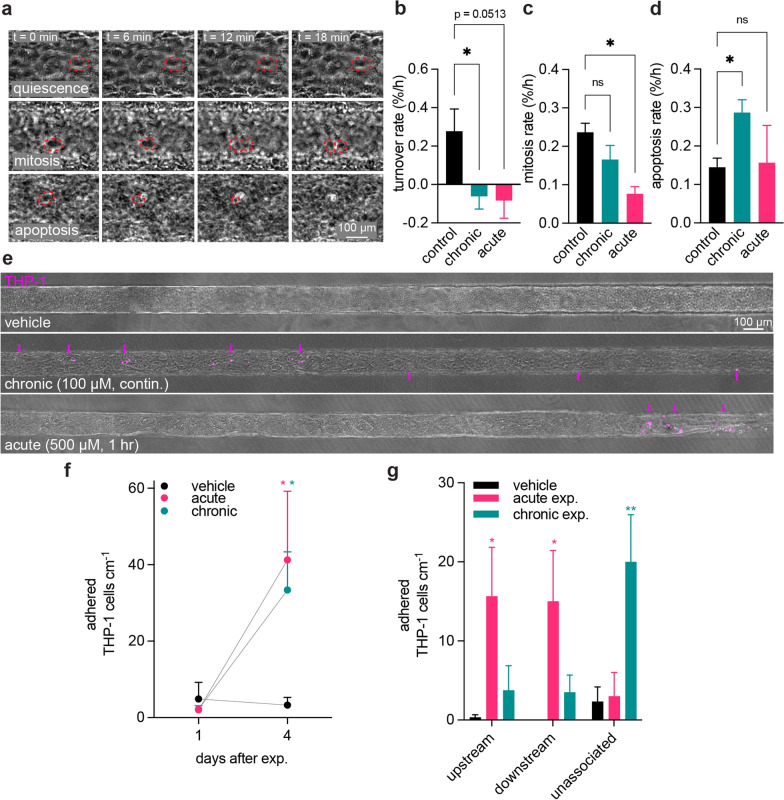
Oxidative stress modulates iBMEC turnover and immune cell adhesion. **a** Representative images of the time course of cell turnover events over an 18-min period. **b–d** Quantification of mitosis rate, apoptosis rate, and turnover, respectively, in response to vehicle, acute oxidative stress, and chronic oxidative stress treatments at day 1 after exposure. *n* = 5 biological replicates for control, *n* = 3 biological replicates for chronic and acute exposure. **e** Representative images of microvessels four days after initial exposure to vehicle, acute, and chronic oxidative stress, where fluorescent THP-1 (magenta) adhesion is indicated by magenta arrows and delaminations are indicated by white arrows. Note that adhered THP-1 cells are completely absent in the vehicle condition. **f** Quantification of adhered THP-1s at days 1 and 4 after initial exposure to oxidative stress. **g** Characterized counts of adherent THP-1 cells according to their distance from a delamination across H_2_O_2_ exposures (up to 150 µm “upstream” of delamination, up to 150 µm “downstream” of delamination, or “unassociated”). Data **f–g** represents *n* = 218 adherent cells across all conditions with *n* = 3 independent microvessels for each condition

We validated RNA sequencing studies by measuring cell turnover and immune cell adhesion in iBMEC microvessels exposed to oxidative stress. First, to determine cell turnover, we manually identified mitotic and apoptotic events from phase contrast images collected over one hour, one day after exposure (Fig. 6a). Overall, the rates of mitosis and apoptosis were approximately equal in control microvessels, resulting in a net turnover rate of 0.037% per hour. However, both chronic and acute oxidative stress exposure resulted in negative cell turnover rates (− 0.062% and − 0.083% per hour, and *p = 0.038 and *p* = 0.051, respectively), indicating a net loss of cells over time (Fig. 6b). Although the turnover rates were similar in response to chronic and acute exposure, the origin of these differences was different. Chronic oxidative stress resulted in a similar mitosis rate to control microvessels, but a higher rate of apoptosis (*p = 0.047) (Fig. 6c). In contrast, acute oxidative stress resulted in a lower rate of mitosis (*p = 0.011), but a similar rate of apoptosis compared to controls (Fig. 6d).

Previously published studies of immune cell behavior in vivo and in vitro primary cell culture have demonstrated a link between inflammatory conditions (such as disease or injury) and increased immune cell recruitment, particularly via adhesion molecules including ICAM-1, VCAM-1, and e-selectin [58–60]. Recent studies in iPSC-derived BBB models found that direct induction of inflammatory conditions with key cytokines can promote upregulation of adhesion molecules and interactions with T cells [61]. To further explore immune cell adhesion in the BBB in the context of oxidative stress as indicated by our transcriptomic results, we perfused microvessels with fluorescently-labeled THP-1 cells, a monocyte-like cell line, one or four days following chronic or acute H_2_O_2_ exposure (Fig. 6e). One day following exposure the vehicle control had 4.90 adhered THP-1s cm^−1^, and there were no statistical differences between the number of adherent immune cells across experimental conditions (*p* = 0.958 for acute exposure, *p* = 0.954 for chronic exposure) (Fig. 6f). However, four days after exposure, the number of adherent immune cells increased to 33.4 cm^−1^ under chronic oxidative stress (*p = 0.032) and 41.3 cm^−1^ under acute oxidative stress (*p = 0.012), while control conditions maintained a low level of immune cell adhesion. Although there was no significant difference in the number of adherent immune cells after four days between chronic or acute exposure, the spatial distribution was dependent on the defect type. Adhesion often occurred in the form of clusters, and predominantly upstream of delaminations (Fig. 6e). This was particularly apparent in acute exposure, which tended to result in more delaminations in comparison to chronic exposure.

To quantify this spatial heterogeneity, each adherent THP-1 was categorized based on its upstream or downstream proximity to a delamination (Fig. 6g). THP-1s were defined as proximal if they were within 150 µm “upstream” or “downstream” of the edge of the delamination; and were defined as “unassociated” if they were further than 150 µm from the delamination. Acute exposure resulted in a majority of adherent immune cells associated (either upstream or downstream) with a delamination (*p = 0.035 vs. control for upstream THP-1s, *p = 0.0395 vs. control for downstream THP-1s). On the other hand, chronic exposure resulted in a majority of adherent immune cells unassociated with a delamination (**p = 0.009 vs. control). This quantification of spatial observations indicates potentially different etiologies of immune cell adhesion in response to different oxidative stress exposures. While structural defects and their influence on immune cell residence time during perfusion may dominate in acute oxidative stress, other modalities must influence the increased immune cell adhesion observed in chronic oxidative stress. While it may be hypothesized that adhesion molecule expression may play a role in chronic exposure, immunofluorescence imaging of ICAM-1 and VCAM-1, critical receptors associated with endothelial cell activation that promote immune cell adhesion, four days after exposure to oxidative stress was inconclusive (Additional file 1: Fig. S9). Future detailed analysis of the spatial distribution of activated endothelial cells and the location of adherent immune cells will be necessary to further determine the influence of oxidative stress on this aspect of immune response.

## Discussion

Oxidative stress plays an important role in the progression of brain diseases, including neurodegeneration and traumatic brain injury [8–11]. Sources of ROS that induce oxidative stress are both intrinsic and environmental, and hence BBB dysfunction is at the nexus of disease progression in response to oxidative stress. In the brain, local oxidative stress generated by parenchymal cellular dysfunction or by toxic proteins can induce BBB dysfunction, which can in turn exacerbate neuroinflammation by entry of blood components [27, 62–64]. Systemic ROS resulting from environmental cues can also damage the BBB, leading to vascular dysfunction, the accumulation of toxic proteins, and downstream neuroinflammation [15–18, 24, 25]. While these effects of oxidative stress have been observed globally, many previous studies have been unable to clearly demonstrate the spatiotemporal manifestation of BBB dysfunction under oxidative stress, as most utilize model organisms that limit this resolution or only study continuous exposure to ROS [25, 27, 28]. Thus, to better understand BBB phenotype during oxidative stress exposure, we assessed the response of 2D monolayers and 3D tissue-engineered microvessels to acute and chronic hydrogen peroxide (H_2_O_2_).

H_2_O_2_ is a ROS that is commonly used in experimental studies of oxidative stress, but dosages must be carefully calibrated, as its relative stability allows it to act as a second messenger of redox signaling, typically at very low concentrations (1–10 nM) [65]. At higher concentrations, H_2_O_2_ is oxidized to more reactive forms, typically the hydroxyl radical that initiates DNA, protein, and lipid damage, leading to oxidative stress [66]. This transition from homeostatic to pathological phenotype was previously studied in 2D monolayers of primary rat BMECs, where three distinct regimes were found: low concentrations from 1 nM to 1 µM H_2_O_2_ which resulted in enhanced angiogenesis, concentrations above 100 µM which resulted in increased permeability, and concentrations above 10 mM which resulted in increased apoptosis [29]. Related studies using a bead assay showed that exposure to < 1 mM H_2_O_2_ enhanced angiogenic responses [67]. Our results for iBMECs in 2D are consistent with these thresholds, as in both chronic and acute profiles, the barrier disruptive effects of H_2_O_2_ were concentration-dependent in a range from 100 µM to 10 mM, which is also within the spectrum of physiological to pathological exposure observed in humans [55, 56].

Comparison of the response of iBMEC monolayers in 2D and 3D is complicated by the differences in flow conditions and the influence of ROCK inhibitor. Both effects sensitize endothelial barriers to H_2_O_2_ exposure. ROCK inhibitor has been demonstrated to exert both protective and detrimental effects on endothelial barrier function [68–73]. The key determinant of phenotype is related to the dual effect of ROCK in facilitating tight junction formation and diminishing local barrier function at sites of actomyosin stress fibers [74, 75]. Barrier function in brain microvascular endothelial cells is derived from tight junctions and iBMECs do not exhibit an extensive network of F-actin stress fibers in response to shear stress. However, oxidative stress may promote stress fiber formation, at least in response to acute exposure, and thus ROCK inhibition may sensitize iBMECs to barrier disruption following oxidative stress exposure (Additional file 1: Fig. S8) [76–80]. Following additional experimentation to model acute and chronic oxidative stress schemes while maintaining 3D iBMEC model viability using ROCK inhibitor, the resultant doses (500 µM for acute exposure, 100 µM for chronic exposure) are on the lower end of the range explored in 2D, but remain aligned with the general regimes of dysfunction explored in aforementioned studies [53–56]. Most importantly, our study of acute exposure to oxidative stress highlights the additional dynamics of antioxidant and recovery processes in a dose-dependent manner, incorporating concentration as well as exposure time, which resulted in unique dysfunctional pathways observed in our 3D tissue-engineered BBB model.

In homeostatic conditions, ROS are continuously generated in cells but are regulated by intrinsic antioxidants and scavengers, key among them superoxide dismutase, catalases, and vitamins [2]. These agents remove or neutralize ROS from their targets and it is when these systems are overwhelmed that the state of oxidative stress, or more specifically, oxidative distress, is reached. The capacity for this counter regulation is site-specific to the tissue and cell type, and the brain vasculature is typically more susceptible to oxidative stress damage due to its many unique sources of ROS [26]. These intrinsic regulatory drivers may account for some of the time-dependent recovery that is reflected in TEER measurements. In acute exposure to H_2_O_2_, there is a brief increase in TEER over 2–4 days after initial exposure, which may account for some antioxidant capacity that rebounds after the removal of the initial perturbation. However, there is a long-term effect of the acute exposure that ultimately reduces TEER (< 500 Ω cm^2^), which may indicate the lag time between the activity of the antioxidant systems and molecular accumulation of ROS beyond the initial capacity.

Oxidative stress induced three types of defects in 3D tissue-engineered iBMEC microvessels: delaminations, focal leaks, and combined focal leak/delaminations. In the human BBB, there are three potential downstream effects demonstrated by these defects: (1) loss of cell–cell junctions, (2) loss of BMEC adhesion to basement membrane, and (3) cell loss due to cytotoxicity. Junctional disruption results in the loss of paracellular barrier function and the leakage of blood components that can contribute to neuroinflammation in brain disease. In the microvessel model, the presence of transient focal leaks identified sites of local junction disruption. In contrast, loss of BMEC adhesion results in small regions of vascular delamination. These delaminations may lead to disrupted local flow patterns that can additionally affect endothelial function, and this downstream effect may be independent or in combination with loss of cell–cell junctions [81]. Lastly, H_2_O_2_ results in cytotoxicity when the dose approaches the limit of complete cell viability in the system—resulting in the most susceptible cells undergoing cell death. In the absence of a wound healing response from the surrounding cells, the loss will similarly result in transient focal leaks [82]. However, further increases in dose will affect a greater population of endothelial cells (as susceptible cells are expected to be randomly distributed, these defects should also be accordingly distributed) that allows cell loss to propagate, forming larger defects.

While all defect types were observed during both acute and chronic oxidative stress, combined focal leak/delaminations were most prevalent under acute conditions. Acute oxidative stress profiles are associated with primary injuries such as traumatic brain injuries or reperfusion following ischemia, which can result in more significant vascular rarefaction [83, 84], of which a primary event can be significant cell loss and inflammation, analogous to the combined focal leak/delaminations reported here. On the other hand, chronic oxidative stress profiles are associated with neurodegenerative diseases that present clinically following accumulation of various sites of damage, typically at the cellular level [85–88]. This difference in defect types observed here emphasizes unique pathways of dysfunction between chronic and acute exposure to oxidative stress, which was supported by bulk RNA-sequencing analysis, followed by functional and protein-level confirmation. Cell turnover is similarly impacted by acute and chronic oxidative stress, but primarily driven by negative regulation of mitosis in acute conditions and by increases in apoptosis in chronic conditions. Immune cell adhesion is increased in both conditions; however, in response to acute oxidative stress, immune cells were more likely to adhere near structural defects, while in response to chronic oxidative stress, most of the adherent immune cells were unassociated with visible defects. This may indicate that the mechanisms for increased immune cell adhesion are different in acute and chronic oxidative stress exposure, and depend on the predominant type of structural defect—acute exposure may initiate increased immune cell adhesion due to changes in fluid dynamics surrounding delaminated defects, while chronic exposure may rely on other etiologies, e.g., adhesion molecule expression. By analogy, plaque build-up is associated with disrupted flow patterns at bifurcations and junctions [89, 90]. Future work in quantifying flow patterns and spatially specific protein-level assessment will be necessary to support this hypothesis, as the local nature of structural defects indicates that global changes may not reflect the true, heterogeneous nature of BBB dysfunction.

Tissue-engineered models of the BBB enable independent control of biological variables, which is important in understanding the systemic, blood-derived impact of oxidative stress in the progression of brain disease and injury. The additional contributions of local, tissue-derived oxidative stress, for example in response to aging or plaque formation in neurodegenerative disease, could be multiplexed with this model in future work to better understand the combined effects of blood- and brain-derived oxidative stress on the BBB. Since co-culture with other cell types and matrix stiffness can alter the characteristics of iBMEC microvessels, we hypothesize that these engineering design choices could also augment the response to oxidative stress (91, 92), which could be explored in future work. Our continued understanding of structural and biological changes to the BBB, which is closely linked with severe disease, will also allow investigation of strategies for recovery or prevention of these changes, with the goal of improving clinical outcomes.

## Conclusions

To match the dynamic nature of oxidant exposure mediated by the BBB during injury and disease, we studied both chronic and acute exposure to H_2_O_2_ and their effect on barrier function and pathological cell processes. The influence of concentration and exposure time highlighted the dose-dependent nature of the response of iBMEC monolayers to oxidative stress. The application of these oxidative stress profiles in 3D microvessel models highlighted the localization of barrier disruption, resulting in distinct, discrete defect categories (focal leaks, delaminations, and combination focal leak/delaminations) with unique distributions based on oxidative stress dose. These were similarly linked to other functional changes under oxidative stress that are unique to each profile, particularly in cell turnover and immune cell adhesion. These functional differences emphasize the importance of appropriate dynamic modeling in the study of disease state or injury. Detailed understanding of the local changes to BBB function under oxidative stress, or other stressors associated with neurodegenerative disease and injury, is key to mapping the multiple etiologies of disease symptomology and designing appropriate interventions to improve outcomes after diagnosis or trauma.

## Supplementary Information

**Additional file 1:** **Figure S1. **Longevity of hydrogen peroxide in cell culture media. **Figure S2. **TEER, cell counts and ROS visualization. **Figure S3. **Dissolved oxygen levels in iBMEC microvessel perfusates. **Figure S4.** Dependence of sensitivity to oxidative stress on baseline TEER values of iBMECs. **Figure S5.** Maintained Lucifer yellow permeability in confluent regions of iBMEC microvessel models exposed to H2O2. **Figure S6.** Expanded RNA-sequencing pathway analysis results. **Figure S7.** Expanded pathway analysis of antioxidant stress genes and reactive oxygen species response genes. **Figure S8.** Stress fiber formation under oxidative stress. **Figure S9.** Adhesion molecule expression in response to oxidative stress. Supplemental materials and methods.

## Data Availability

All data associated with this study are available in the main text or the supplementary materials. The raw data required to reproduce these findings are available from the corresponding author. RNA sequencing data is available in NCBI’s GEO (Accession Number GSE193887).

## Abbreviations

ROS: Reactive oxygen species
H_2_O_2_: Hydrogen peroxide
NDD: Neurodegenerative disease
BBB: Blood–brain barrier
iBMEC: Induced brain microvascular endothelial-like cells
TEER: Transendothelial electrical resistance
PDMS: Polydimethylsiloxane
ROCK: Rho-associated protein kinase
LY: Lucifer yellow
RNA: Ribonucleic acid
DEG: Differentially expressed gene
GSEA: Gene set enrichment analysis
