# Supplementary material for: Effects of acute and chronic oxidative stress on the blood–brain barrier in 2D and 3D in vitro models
Source: Fluids Barriers CNS. 2022 May 12;19:33. doi: 10.1186/s12987-022-00327-x (PMC9097350; doi:10.1186/s12987-022-00327-x)
Supplement: Supplementary file 1 — Additional file 1: Figure S1. Longevity of hydrogen peroxide in cell culture media. Figure S2. TEER, cell counts and ROS visualization. Figure S3. Dissolved oxygen levels in iBMEC microvessel perfusates. Figure S4. Dependence of sensitivity to oxidative stress on baseline TEER values of iBMECs. Figure S5. Maintained Lucifer yellow permeability in confluent regions of iBMEC microvessel models exposed to H2O2. Figure S6. Expanded RNA-sequencing pathway analysis results. Figure S7. Expanded pathway analysis of antioxidant stress genes and reactive oxygen species response genes. Figure S8. Stress fiber formation under oxidative stress. Figure S9. Adhesion molecule expression in response to oxidative stress. Supplemental materials and methods. [file 12987_2022_327_MOESM1_ESM.pdf]

Effects of acute and chronic oxidative stress on the blood-brain barrier in 2D and 3D *in vitro* models

Tracy D. Chung,[1,2] Raleigh M. Linville,[1,2] Zhaobin Guo,[1] Robert Ye,[1,3] Ria Jha,[1,2] Gabrielle N. Grifno,[1,2] Peter C. Searson[1,2,4*]

[1] Institute for Nanobiotechnology, Johns Hopkins University, Baltimore, MD
[2] Department of Biomedical Engineering, Johns Hopkins University, Baltimore, MD
[3] Department of Applied Mathematics and Statistics, Johns Hopkins University, Baltimore, MD
[4] Department of Materials Science and Engineering, Johns Hopkins University, Baltimore, MD

Supplemental Figure S1. Longevity of hydrogen peroxide in cell culture media.
Supplemental Figure S2. TEER, cell counts and ROS visualization.
Supplemental Figure S3. Dissolved oxygen levels in iBMEC microvessel perfusates.
Supplemental Figure S4. Dependence of sensitivity to oxidative stress on baseline TEER values of iBMECs.
Supplemental Figure S5. Maintained Lucifer yellow permeability in confluent regions of iBMEC microvessel models exposed to H_2O_2.
Supplemental Figure S6. Expanded RNA-sequencing pathway analysis results.
Supplemental Figure S7. Expanded pathway analysis of antioxidant stress genes and reactive oxygen species response genes.
Supplemental Figure S8. Stress fiber formation under oxidative stress.
Supplemental Figure S9. Adhesion molecule expression in response to oxidative stress.

Supplemental materials and methods
Hydrogen peroxide detection in cell culture media
Dissolved oxygen detection in iBMEC microvessel perfusate
Heterogeneous permeability of iBMEC microvessels
Immunocytochemistry

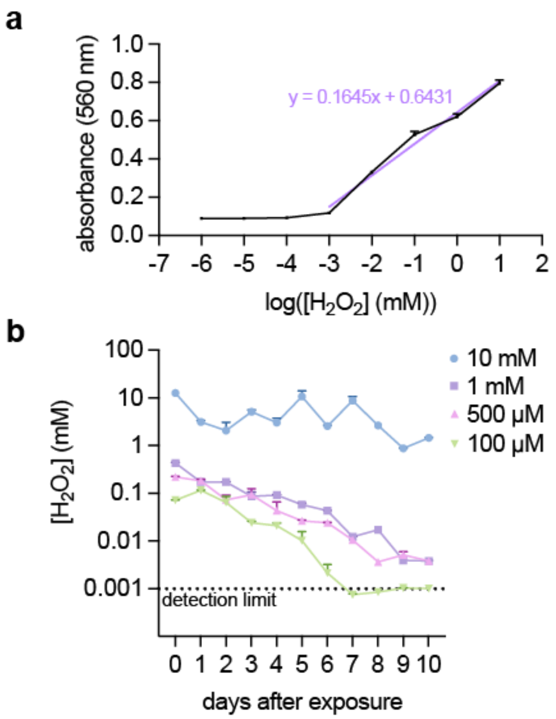

Supplemental Figure S1. Longevity of hydrogen peroxide in cell culture media. (a) Standard curve of various H_2O_2 concentrations in provided reaction buffer and the semilog curve of best fit in the semilog linear regime (line and equation in purple). (b) Degradation of H_2O_2 concentration in cell culture media over ten days. The detection limit of the assay was determined by the sustained minima of all concentrations below 1 μM in the standard curve. n = 3 independent measurements for all conditions.

Summary. This work refers to the H_2O_2 exposure concentration by the H_2O_2-supplemented media prepared on day 0 of experimentation. However, throughout the duration of chronic exposures in particular, H_2O_2 decomposes. High concentrations of H_2O_2 (10 mM) were more stable as commercially-available stock solutions are formulated with stabilizers for long-term storage. As H_2O_2 (and stabilizers) are diluted to lower concentrations, the solutions decompose more dramatically. For both 1 mM and 500 μM concentrations, H_2O_2 levels are relatively stable (within one order of magnitude) for 4-5 days, and then continue to decompose significantly. For 100 μM concentrations, H_2O_2 levels are also relatively stable for 4-5 days, but then drop below the detection limit of the assay at day 7. For the purposes of this study, acute exposures (30 min – 1 h) maintain the true H_2O_2 concentration for its full duration; chronic exposures (7 – 10 days) maintain relatively stable H_2O_2 concentration for several days and remain at measurable levels above blank controls for at least 7 days. This days-long longevity still produces unique functional changes that better recapitulate chronic exposure conditions when compared to acute exposure conditions.

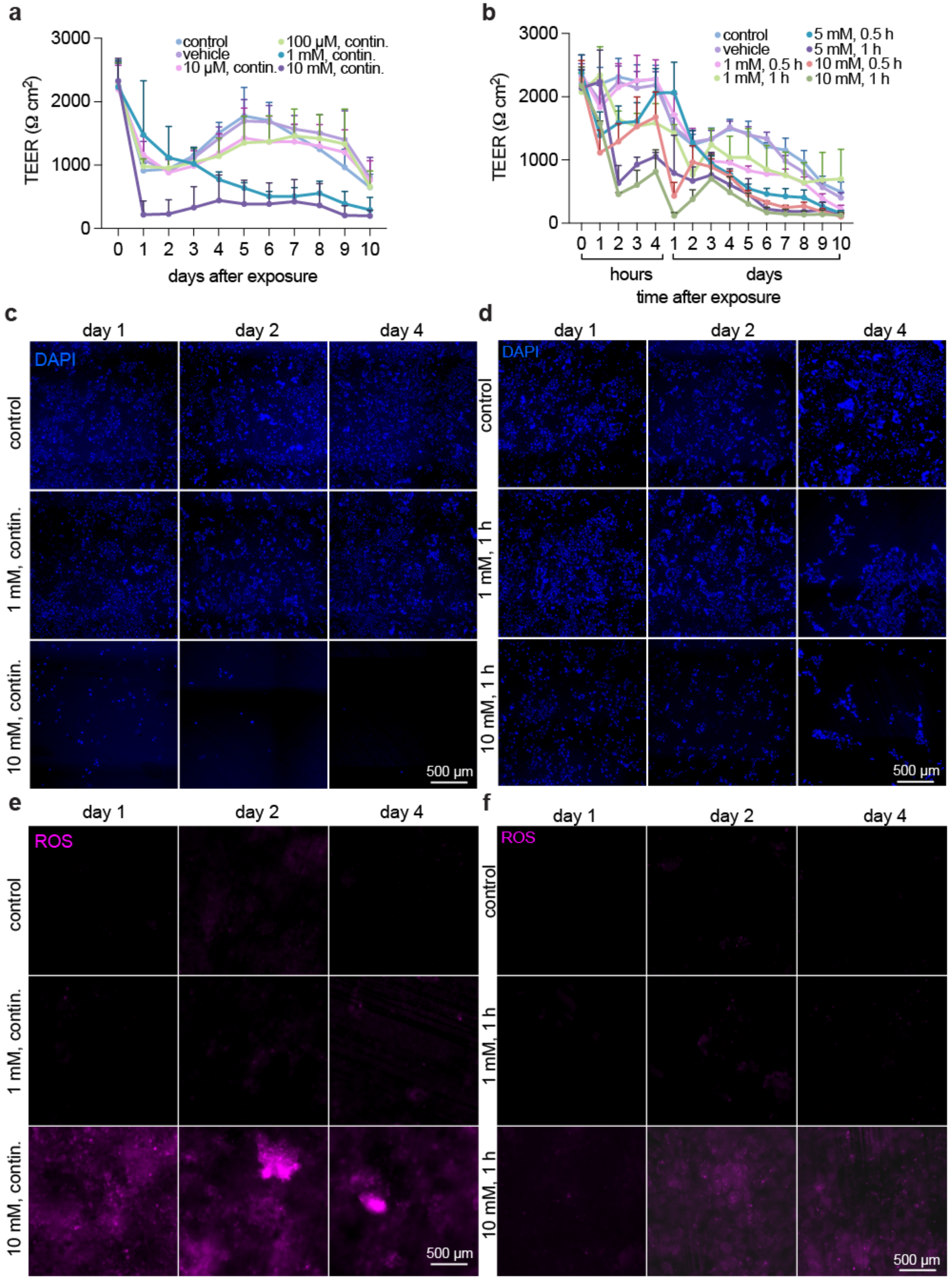

a
b
TEER (Ω cm²)
control
vehicle
10 μM, contin.
100 μM, contin.
1 mM, contin.
10 mM, contin.
days after exposure
5 mM, 0.5 h
5 mM, 1 h
1 mM, 0.5 h
1 mM, 1 h
10 mM, 0.5 h
10 mM, 1 h
hours
days
time after exposure
c
d
e
f
day 1
day 2
day 4
DAPI
ROS
1 mM, contin.
10 mM, contin.
1 mM, 1 h
10 mM, 1 h
500 μm

Supplemental Figure S2. TEER, cell counts and ROS visualization. (a-b) Time course graphical visualization of TEER dynamics following chronic (a) or acute (b) exposure to H_2O_2. Each point represents the mean of three technical replicates across $n = 3$ biological replicates in chronic exposure, and $n = 7$ for acute exposure. (c-d) Representative DAPI images used for cell count quantifications, showing the general trends in decreased cell count with oxidative stress exposure. (e-f) Representative Deep Red ROS assay images used for ROS quantification. Though background membrane fluorescence is increased as cell counts decrease dramatically, quantification accounted for this background quantification and ROS fluorescence was assessed per cell in CellProfiler modules. Brightness was adjusted across all images to a standard LUT range based on visibility of conditions with most fluorescent signal. $n = 3$ biological replicates for both conditions.

Summary. Characterization of responses to acute and chronic H_2O_2 exposure in 2D settings reveal the influence of oxidative stress on cell viability, and the narrow window of exposure that results in measurable, but non-catastrophic biological effects. A wider range of concentrations assessed here assisted in the selection of key exposures defined as acute (ultimately, 10 mM for 1 h in 2D) or chronic (ultimately, 1 mM continuously). The TEER and cell loss dynamics observed in 2D were also used as comparisons to evaluate analogous acute and chronic 3D exposures.

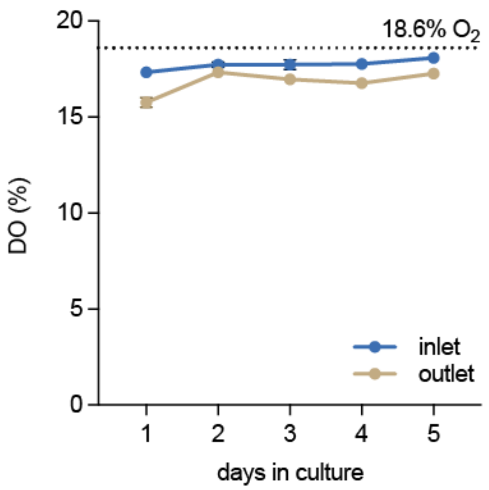

Supplemental Figure S3. Dissolved oxygen levels in iBMEC microvessel perfusates. (a) Measured dissolved oxygen (DO) levels in the inlet and outlet iBMEC basal media reservoirs over several days. Each measurement was temperature-compensated. n = 2 biological replicates.

Summary. To ensure that oxygen depletion during perfusion of the microvessels did not contribute to oxidative stress, we measured the levels of dissolved oxygen in the medium in the inlet reservoir and in the perfusate from the outlet over 5 days using a dissolved oxygen probe (RCYAGO, DO9100). The dissolved oxygen in the inlet reservoir was on average 17.7%, close to the expected value of 18.6% under normal cell culture conditions (5 vol.% CO_2 and 37 ˚C) (Newby et al., Placenta, 2005). The concentration in the outlet perfusate was on average 16.8%, indicating a ~ 0.9% drop in dissolved oxygen due to cell metabolism during perfusion. These results confirm that the conditions in the microvessel do not result in hypoxic conditions in the microvessel.

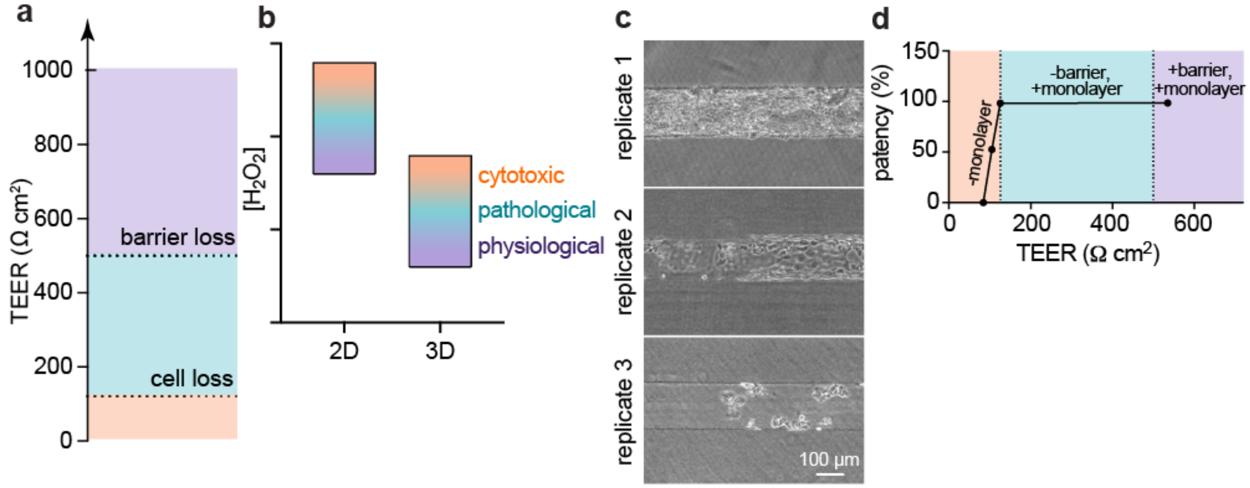

Supplemental Figure S4. Dependence of sensitivity to oxidative stress on baseline TEER values of iBMECs. (a) Schematic of the transition points between physiological homeostasis, initiation of barrier loss, and initiation of cell loss based on previous publications and our studies of cell counts. (b) Schematic cartoon demonstrating the expected differences between 2D and 3D H_2O_2 responses, due to innate differences in dimensionality, flow profile, and chemical reagents used in 3D models. (c) Representative phase images of three different biological replicates of iBMECs seeded into 3D microvessel constructs resulting in vastly different patency after 24 h. (d) Percent coverage of microvessels after 24 h is dependent on the initial TEER values (measured 48 h following seeding on transwell supports for measurement) of the biological replicate based on differentiation round.

Summary. Transitioning chronic and acute oxidative stress exposures in 3D iBMEC microvessels revealed higher sensitivity that were primarily dependent on inherent initial TEER of each iBMEC differentiation and the use of ROCK inhibitor (explored in Fig. 3b). Utilizing measures of barrier (TEER) and cell monolayer viability (patency of produced microvessels), we identified three regimes of oxidative stress response: no measurable change in barrier of monolayer patency, a decline in barrier function with no measurable change in monolayer patency, or a decline in barrier function and monolayer patency. To study the measurable effects of chronic and acute oxidative stress, we must identify doses of H_2O_2 that recapitulate a measurable change in barrier function with little to no cell loss.

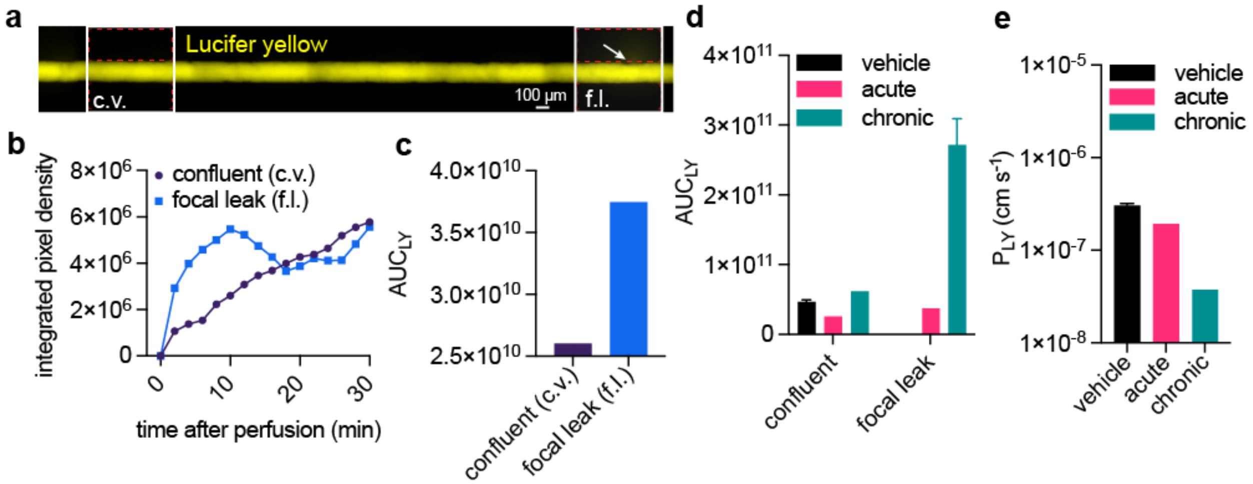

Supplemental Figure S5. Maintained Lucifer yellow permeability in confluent regions of iBMEC microvessel models exposed to H_2O_2. (a) Representative image of a microvessel after initiation of perfusion with 200 μM Lucifer yellow. White arrow indicates dye leakage at a focal leak, white boxes indicate regions for further permeability analysis representing a confluent vessel region (c.v.) and a focal leak (f.l.), red dotted boxes indicate regions of interest that define the extracellular matrix (ECM) from which dye permeation was calculated. (c) Raw integrated pixel density plots over the time period of epifluorescent imaging in the above confluent vessel region (c.v.) and focal leak region (f.l.). (d) Calculated areas under the curve (AUC) of Lucifer yellow perfused iBMEC microvessel regions corresponding to (b-c). (e) Calculated areas under the curve (AUC) of Lucifer yellow perfused iBMEC microvessel in confluent and focal leak regions across H_2O_2 exposure conditions. (f) Local Lucifer yellow permeability values in confluent regions in iBMEC microvessel models exposed to acute and chronic oxidative stress and corresponding vehicle control. *n* = 2 biological replicates for all conditions, with two distinct regions assessed in each replicate.

Summary. To emphasize the influence of discrete, local defects over global paracellular permeability changes, we conducted segmented analysis to conclude that the permeability in no-defect regions of iBMEC microvessel models are similar (~ 1 x 10^{-7} cm s^{-1}) across oxidative stress conditions and corresponding controls. In bulk permeability measurements, these discrete regions are averaged and result in markedly higher permeability in both chronic and acute oxidative stress conditions (~ 1 x 10^{-5} cm s^{-1}). We assert that local analysis more accurately represents the true paracellular permeability of perfused dyes and the dominance of local defects in response to chronic and acute oxidative stress.

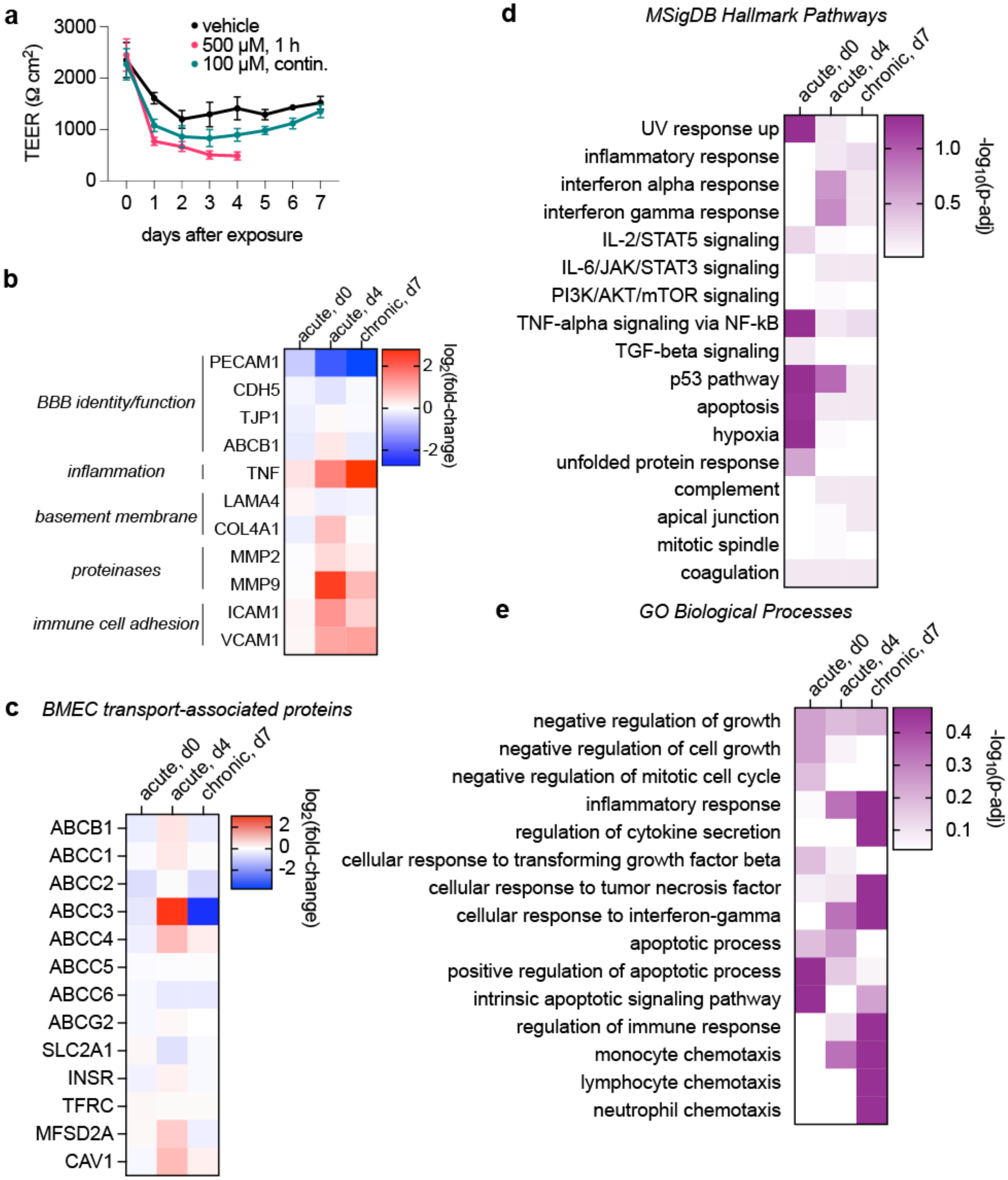

Supplemental Figure S6. Expanded RNA-sequencing pathway analysis results. (a) TEER time-course of iBMEC samples used for RNA-sequencing. (b) Heatmap indicating fold-changes from their corresponding vehicle control of genes of interest related to BBB identity/function, inflammation, basement membrane, proteinases, and immune cell adhesion. (c) Heatmap indicating fold-changes from their corresponding vehicle control of genes associated with transport systems of BMECs. (d-e) Heatmaps of adjusted p-value of expanded selection of pathways of GO Biological Processes and MSigDB Hallmark Pathway terms, respectively, from statistically

significant DEGs and genes with $\log_2$(fold-change) > 3. $n = 4$ biological replicates for all conditions.

Summary. Expanded analysis of transcriptomic changes following chronic or acute oxidative stress emphasize the importance of cell viability and inflammatory responses. Additional pathways and functions for future study are also suggested by these results, particularly changes to endothelial function (**Supp. Fig. S6b**), efflux pump activity (**Supp. Fig. S6c**), and TNFα-mediated inflammation (**Supp. Figs. S6d,e**).

Key gene symbols and translated protein:
ABCB1 – P-glycoprotein
ABCC family – multidrug resistance protein family
SLC2A1 – facilitated glucose transporter 1
INSR – insulin receptor
TFRC – transferrin receptor

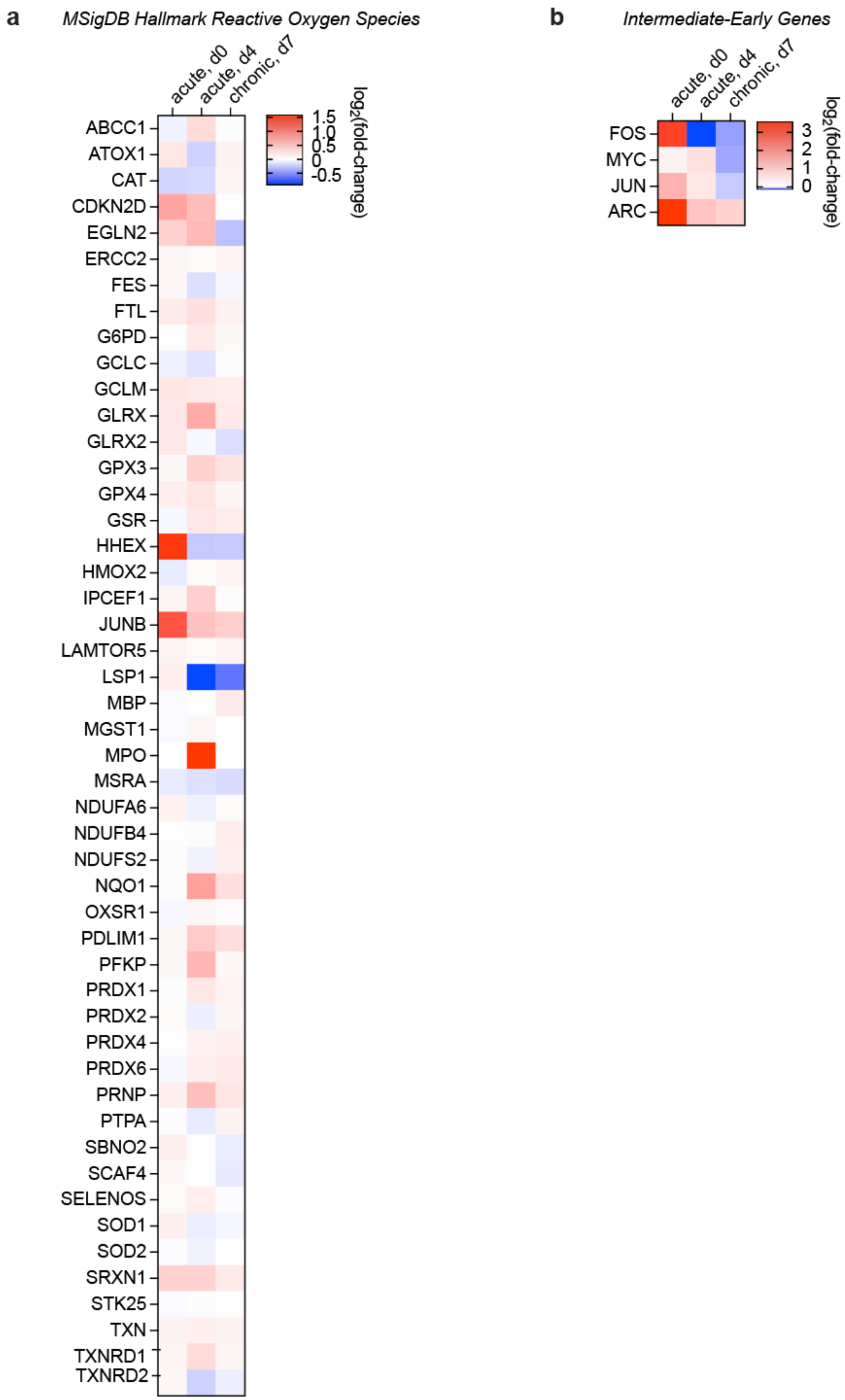
a
MSigDB Hallmark Reactive Oxygen Species
acute, d0
acute, d4
chronic, d7
ABCC1
ATOX1
CAT
CDKN2D
EGLN2
ERCC2
FES
FTL
G6PD
GCLC
GCLM
GLRX
GLRX2
GPX3
GPX4
GSR
HHEX
HMOX2
IPCEF1
JUNB
LAMTOR5
LSP1
MBP
MGST1
MPO
MSRA
NDUFA6
NDUFB4
NDUFS2
NQO1
OXSR1
PDLIM1
PFKP
PRDX1
PRDX2
PRDX4
PRDX6
PRNP
PTPA
SBNO2
SCAF4
SELENOS
SOD1
SOD2
SRXN1
STK25
TXN
TXNRD1
TXNRD2
1.5
1.0
0.5
0
-0.5
log2(fold-change)
b
Intermediate-Early Genes
acute, d0
acute, d4
chronic, d7
FOS
MYC
JUN
ARC
3
2
1
0
log2(fold-change)

Supplemental Figure S7. Expanded pathway analysis of antioxidant stress genes and reactive oxygen species response genes. (a) Heatmap of MSigDB Hallmark Reactive Oxygen Species pathway constituent genes and corresponding log_2(fold-change) values for each oxidative stress condition. (b) Heatmap of Intermediate-Early Genes (IEGs) and corresponding log_2(fold-change) values for each oxidative stress condition.

Summary. Expanded analysis of transcriptomic changes involved in canonical oxidative stress and reactive oxygen species genes reveals general upregulation of many genes, although the combined score significance of the pathway as a whole is not statistically significant. Additionally, multiple Intermediate-Early Genes that are rapidly transcribed following cellular perturbation are heavily upregulated immediately following oxidative stress exposure, with waning effects as time from initiation increases.

Key gene symbols and translated protein:
ABCC1 – multidrug resistance associated protein 1
CAT – catalase
GLRX family – glutaredoxin family
GPX family – glutathione peroxidase family
PRDX family – peroxiredoxin family
SOD family – superoxide dismutase family
TXN – thioredoxin
FOS – c-Fos/AP-1
MYC – c-Myc
JUN – c-Jun/AP-1
ARC – activated regulated cytoskeleton associated protein

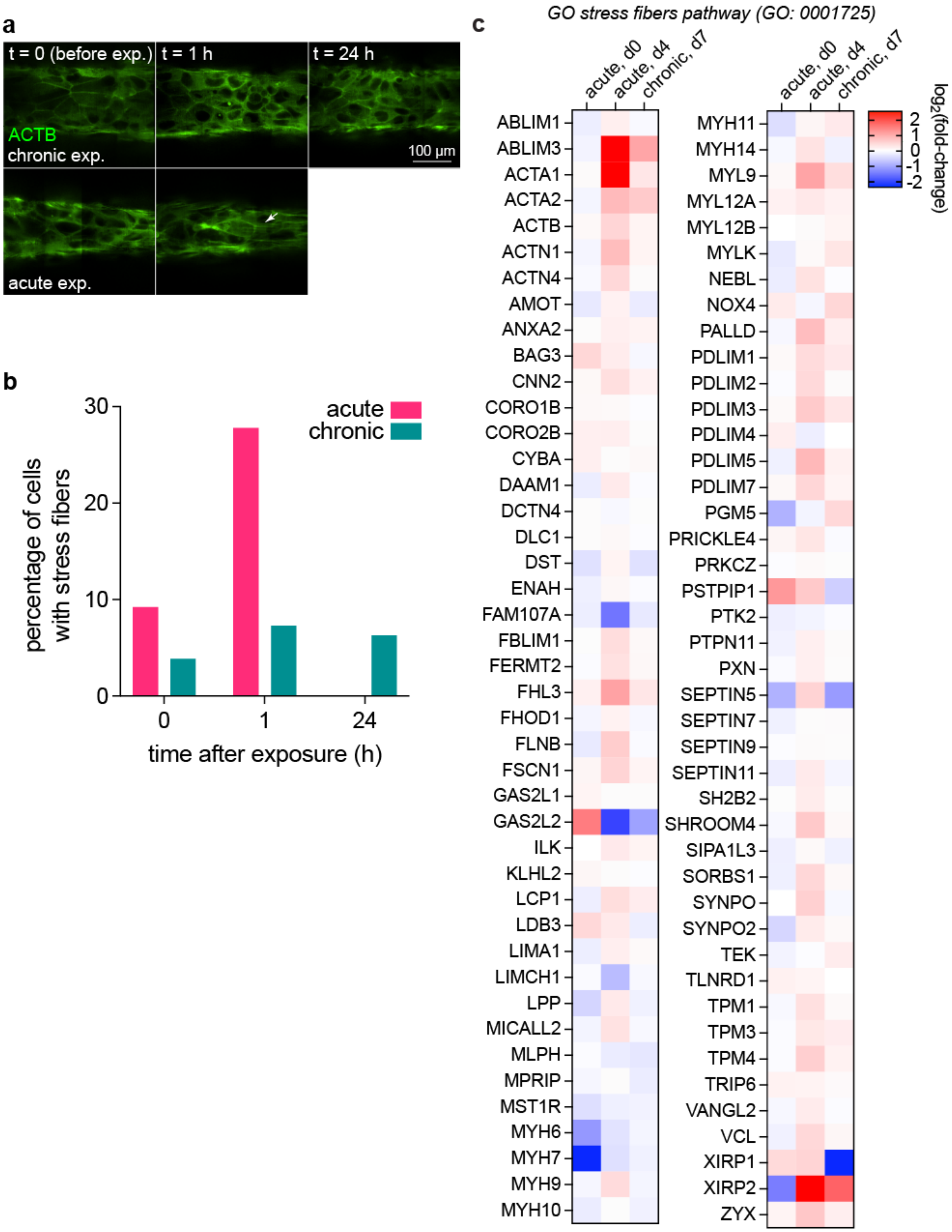

Supplemental Figure S8. Stress fiber formation under oxidative stress. (a) Representative images of native ACTB fluorescent tags before and after acute (500 µM for one hour) and chronic (100 µM continuously) H_2O_2 exposure. (b) Quantification of the percentage of cells with fiber-like

ACTB localization across the polar planes of iBMEC microvessels after acute and chronic H_2O_2 exposure. *n* = 1 biological replicate. (c) Heatmap of GO stress fibers pathway (GO: 0001725) constituent genes and corresponding log_2(fold-change) values for each oxidative stress condition.

Summary. Following acute oxidative stress, stress fibers in ACTB-tagged iBMEC microvessels are visible primarily in the short-term (1 h) after intense, acute H_2O_2 exposure (500 μM), and are minimally visible following milder, chronic H_2O_2 exposure (100 μM). Previous work with iBMEC elucidated a sparse network of stress fibers under shear stress in comparison to immortalized endothelial cell lines, which may indicate a minimal reliance on these fibers to maintain shape and cell-cell junctions under stress. However, under oxidative stress, the formation of stress fibers may be associated with the formation of structural defects (see Fig. 4), which is a potential future direction of this work.

Key gene symbols and translated protein:
ABLIM1 – actin binding LIM protein 1
ACTA family – actin alpha family
ACTB – actin beta
ACTN family – actinin family
ANXA2 – annexin A2
CNN2 – calponin 2
FLNB – filamin B
MYH family – myosin heavy chain family
MYL family – myosin light chain family
PXN – paxillin

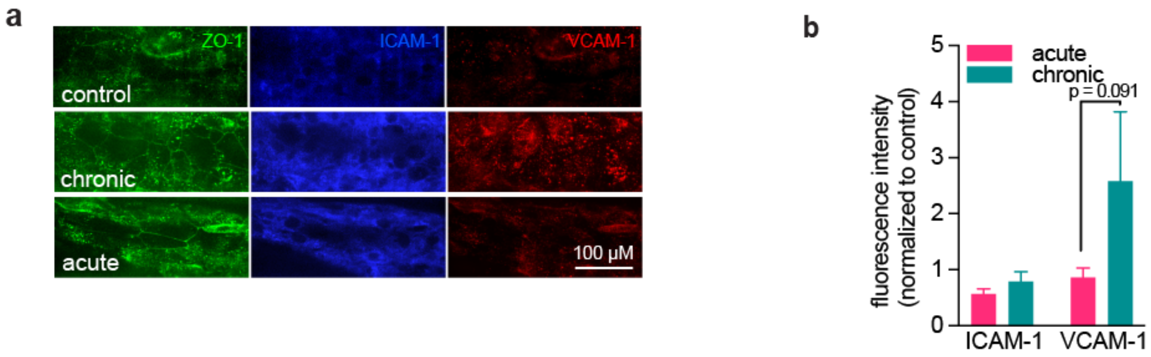

Supplemental Figure S9. Adhesion molecule expression in response to oxidative stress. (a) Representative confocal images of microvessels with native ZO-1 fluorescent tags, and stained ICAM-1 and VCAM-1 in control conditions, chronic exposure, and acute exposure. (b) Quantification of fluorescence intensity for ICAM-1 and VCAM-1 in microvessels at day 7 after initiation of chronic and acute oxidative stress. Values reflect the average integrated pixel density of maximum projection z-stacks at two distinct regions in each microvessel normalized to a time-matched control. Bars represent the mean of two distinct imaging regions across n = 3 biological replicates for both conditions.

Summary. Following immune cell adhesion experiments (see Fig. 6e-g), we observed and quantified an association between adherent immune cells and visible delamination defects, particularly in acute H_2O_2 exposure which tend to produce more delamination defects, while most adherent immune cells in chronic exposure were unassociated with visible defects. We hypothesize that canonical immune cell adhesion molecule expression may play a unique role in immune cell adhesion in chronic H_2O_2 exposure. Although the immunocytochemistry shown here is inconclusive, further study using more specific spatial alignment between discrete defects, adhered immune cells, and adhesion molecule protein expression may establish these correlations in a more robust manner.

Supplemental materials and methods

Hydrogen peroxide detection in cell culture media

To measure the longevity of hydrogen peroxide over the length of oxidative stress exposure experiments, solutions of 0 M (blank control), 100 µM, 500 µM, 1 mM, and 10 mM H_2O_2-supplemented basal iBMEC cell culture media were generated and maintained in a cell culture incubation chamber. H_2O_2 levels were measured using the Amplex Red Hydrogen Peroxide Kit (Thermo Fisher, A22188) by adding a working solution of the kit with horseradish peroxidase and the Amplex Red reagent to each sample (and corresponding negative and positive controls in provided reaction buffer) and measuring the absorbance at 560 nm after 30 minutes of incubation at 37 ˚C. Absorbance readings were extrapolated to concentration measurements based on a standard curve of H_2O_2 concentrations in provided reaction buffer.

Dissolved oxygen detection in iBMEC microvessel perfusate

Oxygen levels in the inlet and outlet of iBMEC microvessel model media reservoirs were measured by an electrochemical dissolved oxygen (DO) probe with temperature compensation. (RCYAGO, DO9100). The outlet reservoir of iBMEC microvessel models were replaced with Tygon tubing (Tygon, LMT-55) that dispensed media perfusate into a glass Erlenmeyer flask with a rubber stopper (PYREX, Corning, 498025). The DO probe was calibrated to 21% in air and to 0% in deoxygenated water and then used to measure both media volumes. Due to humidification of cell culture incubators, the actual oxygen percentage available for exchange in incubators in 18.6%, as opposed to the typical 21% O_2 in air.

Heterogeneous permeability of iBMEC microvessels

To demonstrate the discrete, local nature of defects in response to oxidative stress, and the heterogeneity of apparent permeability that follows, sectioned permeability analysis was conducted across microvessel conditions (vehicle, chronic oxidative stress via 100 µM H_2O_2 exposure, and acute oxidative stress via 500 µM H_2O_2 exposure for one hour). 2 µM Alexa Fluor647-conjugated 10 kDa dextran (Thermo Fisher Scientific, cat. no. D22914) and 200 µM Lucifer yellow (LY; Sigma, CH dilithium salt) solutes in BBB maintenance medium were perfused through each microvessel. Microvessels were then imaged (Nikon Eclipse TiE) at 10x magnification and maintained in an environmental chamber at 37 ˚C. Epifluorescence illumination was controlled by X-Cite 120LEDBoost (Excelitas Technologies). Phase contrast images (assembled from images from 10 adjacent frames) were acquired every two minutes at the longitudinal top plane, midplane, and bottom plane of the microvessel; fluorescence images were acquired every two minutes at the microvessel midplane. Microvessels were imaged for 10 minutes before and 1 hour following perfusion with the fluorescent solutes.

To calculate bulk permeability, images were cropped (ImageJ, NIH) to a central 6 mm x 0.6 mm rectangle and sectioned into 10 regions of interest, each 0.6 mm x 0.6 mm. The integrated pixel density was plotted over time for each ROI and the permeability (cm s^{-1}) was calculated using $P=(r/2)(1/\Delta I)(dI/dt)$, where r is the vessel radius, ΔI is the increase in fluorescence intensity upon

initiation of perfusion of the solute, and dI/dt is the rate of fluorescence intensity increase as the solute permeates into the hydrogel. The average permeability was calculated via linear least squares fits of dI/dt over 60 minutes. To calculate local permeability, two distinct regions (with or without a visible focal leak) were cropped to an area of 0.6 mm x 0.6 mm. The integrated pixel density in the microvessel lumen and the surrounding ECM regions were separately measured and the permeability was calculated using the same equation as above, where ΔI was the increase in fluorescence intensity in the lumen only upon initiation of perfusion of the solute, and dI/dt was the rate of fluorescence intensity increase in the ECM regions only as the solute permeates into the hydrogel. Additionally, areas under the curve (AUC) of raw integrated pixel density versus time were estimated using trapezoidal integration.

Immunocytochemistry

Immunocytochemistry was performed seven days after initial exposure to H_2O_2. Briefly, microvessels were perfused and washed with cold DPBS (Invitrogen, 14080055) for five minutes, then fixed with 3.7% paraformaldehyde solution overnight at 4 ˚C. Microvessels were then stained by: (1) blocking and permeabilizing with blocking solution composed of 0.2% Triton-X 100 (Sigma-Aldrich, T9284) and 10% normal goat serum (Cell Signaling Technology, 5425S) overnight at 4 ˚C, (2) perfusing with primary antibody solution for 4 hours at room temperature, (3) washing with blocking solution overnight at 4 ˚C, (4) perfusing with secondary antibody solution for 30 minutes at room temperature, and (5) washing with blocking solution overnight at 4 ˚C before imaging. Primary antibodies were all against human proteins, including ICAM-1 (Abcam, ab2213) and VCAM-1 (Abcam, ab134047). Confocal z-stacks (0.4 µm in thickness) were acquired at 40x magnification on a swept field confocal microscope system (Prairie Technologies) and illumination was provided by an MLC 400 monolithic laser combiner (Keysight Technologies). Reconstruction of microvessels were assembled from approximately 400 slices.
